# Cancer-Causing Mutations Alter the Interplay Between Loop Dynamics and Catalysis in the Protein Tyrosine Phosphatases SHP-1 and SHP-2

**DOI:** 10.64898/2026.03.02.708844

**Published:** 2026-03-03

**Authors:** Alfie-Louise R. Brownless, Michael Robinson, Shina C. L. Kamerlin

**Affiliations:** 1 School of Chemistry and Biochemistry, Georgia Institute of Technology, 901 Atlantic Drive NW, Atlanta, Georgia 30332, USA; 2 Department of Chemistry – BMC, Uppsala University, BMC Box 576, S-751 23 Uppsala, Sweden; 3 School of Chemical and Biomolecular Engineering, Georgia Institute of Technology, 311 Ferst Drive, Atlanta, GA 30332, USA; 4 Department of Chemistry, Lund University, Box 124, 221 00 Lund, Sweden

**Keywords:** SHP-1, SHP-2, Protein Tyrosine Phosphatases, Loop Dynamics, Allostery, Oncogenic Mutations

## Abstract

The protein tyrosine phosphatases (PTPs) SHP-1 and SHP-2 play complex roles in a variety of signaling pathways, including those involved in cancers and other diseases, making them important drug targets. These two PTPs have superimposable active sites, but different biological functions *in vivo*, including opposing roles in cancer development. Unique to these PTPs is the presence of two tandem Src homology 2 (SH2) domains, which regulate access to the phosphate binding site in the catalytic domain, through an autoinhibition mechanism. Studies of the allosteric regulation and dynamics of these PTPs, as well as associated drug discovery efforts, typically focus on autoinhibition rather than the dynamics of a catalytic loop in the phosphatase domain, the WPD-loop, which is essential for PTPase activity. However, recent deep mutational scanning data has demonstrated that oncogenic mutations also regulate WPD-loop motion in SHP-2. We provide here a detailed computational study of WPD-loop dynamics and catalysis in wild-type and mutant full-length and truncated (catalytic domain only) SHP-1 and SHP-2, demonstrating that many oncogenic residues lie on the allosteric pathways regulating WPD-loop dynamics. Mutations at these positions alter WPD-loop dynamics, disrupting the active site and negatively impacting catalysis. Further, our simulations provide molecular insight into the link between the presence of the SH2 domains and loop motion in the catalytic domain, and, importantly, how it differs between the two PTPs. Taken together, our work showcases the impact of altered WPD-loop motion in oncogenic SHP-1 and SHP-2 variants, opening new strategies for selectively targeting these important therapeutic enzymes.

## Graphical Abstract

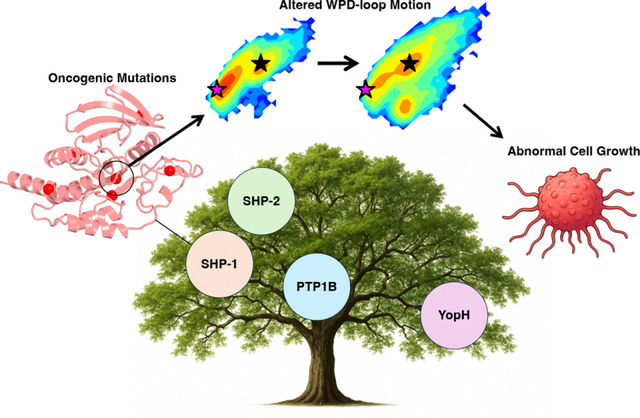

## Introduction

Protein tyrosine phosphatases (PTPs) are a large family of enzymes^1^ that regulate cellular signaling,^2^ thus frequently playing major roles in disease-related pathways.^3^ PTPs act in tandem with protein tyrosine kinases (PTKs) to phosphorylate and dephosphorylate their substrates,^4^ allowing for signal propagation across crucial molecular pathways. Both PTPs and PTKs play complex roles in regulating signaling. However, while wild-type PTKs exhibit positive effects on cell proliferation, PTPs can exhibit either positive or negative roles in proliferation, and are thus more variable in their roles in disease progression.^5^ Furthermore, due to their central roles in biology, aberrations in PTP function have been linked to human cancers, diabetes, autoimmune disorders, cardiovascular disorders, metabolic diseases, and neurological diseases.^3, 4, 6–10^ This makes PTPs crucial (but elusive) drug targets.^11–13^

**Figure 1.**
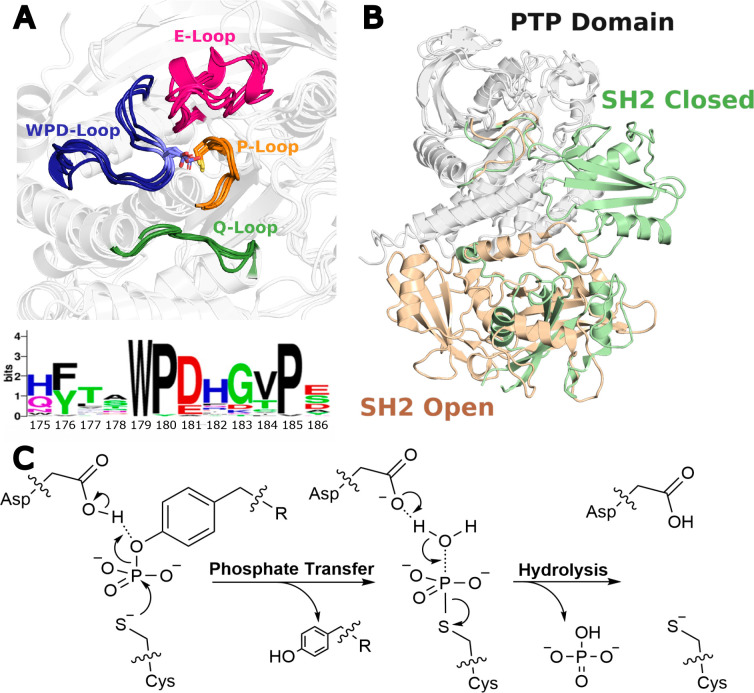
(**A**) Visual alignment of the PTP1B, SHP-1, SHP-2, and YopH active sites with key active site loops emphasized as follows: WPD-loop (blue), P-loop (orange), Q-loop (green), E-loop (pink). Shown below this are the sequence similarities of the WPD-loop across a collection of PTPs, taken from ref. ^27^. (**B**) Overlay of the structure of SHP-1 with its Src homology 2 (SH2) domains in the catalytically active open conformation (tan) (PDB ID: 3PS5^28^), and in the autoinhibited closed conformation (green) (PDB ID: 2B3O^29^). (**C**) Conserved two-step catalytic mechanism of PTPs, proceeding through acid-base catalysis involving an aspartic acid side chain located on the WPD-loop. The second (hydrolysis) step of this process has been shown to be rate-limiting in analogous PTPs.^30–32^ The WebLogo^33^ in panel (**A**) was originally presented in ref. ^27^ under a CC-BY 4.0 license. The catalytic mechanism in Panel C is adapted from ref. ^23^, again published under a CC-BY license. Both images copyright © The Authors. Published by the American Chemical Society.

A major challenge with targeting PTPs is the fact that their core catalytic (PTP) domain is highly conserved.^14^ Further, all PTPs share highly conserved active sites including a conserved phosphate-binding loop (P-loop, C(X)_5_R) motif,^15^ which makes inhibitor selectivity a particular challenge.^16, 17^ However, PTPs also carry a conserved mobile loop, the WPD-loop, which undergoes a substantive conformational change from catalytically “open” to a catalytically “closed” conformation.^18, 19^ In doing so, they position an (again) conserved aspartic acid side chain into the active site, that acts as a general acid/base in the two-step reaction catalyzed by PTPs (Figure 1).^19^ Importantly, despite the high conservation of the catalytic domain, active site structure, key catalytic loops, and chemical mechanism, the catalytic rates of PTPs vary by several orders of magnitude.^20^ This has been argued to be driven by differences in WPD-loop dynamics across PTPs,^21–23^ making the allosteric regulation of the WPD-loop of PTPs an attractive target for novel inhibitors that act by blocking proper closure of the WPD-loop into a catalytically competent position.^24–26^ Achieving this requires intimate understanding of the allosteric regulation of WPD-loop motion in the PTP of interest, and how this regulation is modified by disease-causing mutations.

The focus of this work is on the role of oncogenic mutations on regulating WPD-loop motion (and thus activity) in the protein tyrosine phosphatases SHP-1 and SHP-2. Both enzymes have complicated relationships to cancer signaling, as both SHP-1 and SHP-2 can act as both oncogenes and as tumor suppressors, depending on biological context.^7, 34–38^ However, their prominent roles in oncogenesis makes them attractive anticancer drug targets.^10, 35, 37, 39, 40^ Interestingly, and uniquely to these enzymes, SHP-1 and SHP-2 are the only cytosolic PTPs to express two tandem Src homology 2 (SH2) domains,^41^ which are involved in autoinhibition, regulating access to the phosphate binding site in the catalytic domain (Figures 1B and S1).^28, 29, 42–44^ Curiously, despite the fact that SHP-1 and SHP-2 are an evolutionary pair of enzymes,^19^ with both high sequence conservation (59% overall, 60% across their catalytic domains) and structural conservation,^14^ the impact of the SH2 domains on each enzyme is very different. In SHP-1, the presence of the SH2 domains primarily impacts *K*_M_ rather than *k*_cat_ as shown by comparison to the activity of the truncated phosphatase domain.^30^ In contrast, in SHP-2, the SH2 domains also have a significant impact on *k*_cat_,^45^ suggesting a difference in active site loop dynamics. We note that in both SHP-1 and SHP-2, the C-terminal tail also plays an important role in regulation.^41^

Understanding the origins of these differences in catalytic loop dynamics, how they are regulated by the SH2 domains in the two enzymes, and how they are impacted by oncogenic mutations is an important step towards being able to develop selective inhibitors preferentially targeting only one of the two enzymes. To this end, we initiate our study with a comparison of the WPD-loop dynamics of the SHP-1 and SHP-2 PTPs to that of the WPD-loops of the much better studied enzymes PTP1B and YopH,^3, 11, 20, 22, 23, 31, 32, 46–54^ and show that WPD-loop dynamics in SHP-1 and SHP-2 are different not just between the two enzymes but also different from that of PTP1B and YopH. We then demonstrate that, consistent with kinetic measurements,^30, 45^ from a conformational perspective, the SH2 domains have a differential impact on WPD-loop dynamics of full-length SHP-1 versus their impact in SHP-2, despite sequence similarities across both the catalytic and SH2 domains. Finally, we present a detailed computational comparison of WPD-loop dynamics in truncated catalytic domain forms of SHP-1 and SHP-2, focusing on both wild-type and oncogenic variants of each enzyme. We further leverage empirical valence bond theory to characterize the impacts of chosen pathogenic variants’ chemical barriers in the rate-limiting step of the generalized PTP mechanism. We note that in both enzymes, both the distributions of loop conformations and reaction barriers are shifted in the pathogenic variants. Taken together, our work shows the sensitivity of WPD-loop motion across PTPs in particular to WPD-loop sequence, resulting in significant differences in activity and regulation of these enzymes. This shows how nature can use a simple structural motif to regulate catalysis in a broad range of biological contexts and provides an important target for future drug discovery efforts.

## Results and Discussion

### Comparison of WPD-loop Dynamics Across Wild-Type SHP-1, SHP-2, PTP1B and YopH

SHP-1 and SHP-2 share a high degree of sequence similarity – 59% overall primary sequence identity, as well as 60% similarity in their catalytic domains,^14^ having evolved as an evolutionary pair.^19^ This is much higher than the sequence identity, for instance, between SHP-1 and PTP1B (38%) or YopH (20%), or between SHP-2 and PTP1B (39%) or YopH (20%, all values calculated using MUSCLE,^55^ aligning on the catalytic domain). Given the high sequence similarity between SHP-1 and SHP-2, and the fact that PTPs tend to have very highly structurally conserved catalytic domains overall,^14^ it would stand to reason that SHP-1 and SHP-2 would have WPD-loop dynamics that are similar to that of each other, but different (potentially) to that of PTP1B and YopH. However, there are differences in both the sequences of the WPD-loop and adjacent residues in these PTPs (Figure 1A), which could lead to different WPD-loop dynamics, and perhaps should be expected from the observation that SHP-1 and SHP-2 have distinct non-redundant biological functions.^19^

To address this question, as our starting point, we have compared the dynamical behavior of the truncated catalytic domains of SHP-1 and SHP-2, in the absence of any autoinhibition from the SHP-1/2 SH2 domains. We have performed simulations of each enzyme initiated from both the WPD-loop open and closed states of each of the unliganded and phosphoenzyme intermediate states of each enzyme, as outlined in the Materials and Methods. Our choice of simulating the phosphoenzyme intermediate rather than the Michaelis complex is due to the fact that the hydrolysis of this intermediate (Figure 1C) is the rate-limiting step of catalysis in PTP1B,^32^ YopH^31^ and SHP-1,^30^ and thus loop-dynamics at this intermediate (and the ability to maintain a catalytically competent closed conformation of the loop) are crucial for function. Finally, we note an aside that there exist variations in residue numbering for SHP-1 based on different interpretations of sequence.^27, 56–58^ Throughout this paper we use the residue numbering used in the COSMIC database,^59^ among other sources, for consistency.

Figure S2 shows a comparison of the difference in C_α_-atom root mean square fluctuations (RMSF) during our molecular dynamics simulations of SHP-1 and SHP-2 (8 × 1.5 μs replicas simulated per enzyme), obtained by calculating the RMSF for each residue in each enzyme. Interestingly, we observe relatively similar overall flexibility profiles for the two enzymes (note that RMSF measures local and not global flexibility), with the exception of two regions, around residues 294–298 and 314–321, with reduced mobility in SHP-1 compared to SHP-2. These peaks correspond to protein loops distal to the active site, that exist in both SHPs, but take on a more extended form in SHP-2 than in SHP-1 (Figure S2). Beyond these differences, our RMSF analysis suggests that the overall flexibility of the isolated catalytic domain is very similar in the two enzymes with almost all corresponding residues exhibiting a similar RMSF.

**Figure 2.**
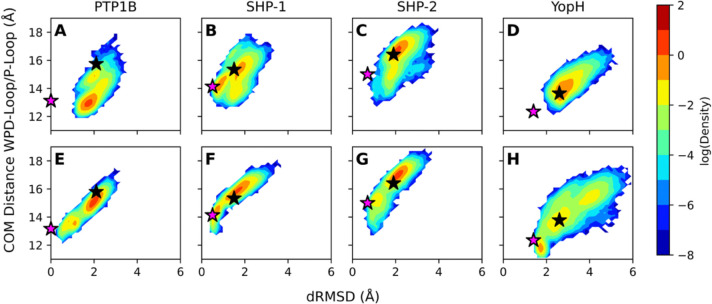
2D histograms of the distance root mean square deviations (dRMSD, Å) of the distances between the C_α_-atoms of all WPD-loop and all P-loop residues in wild-type (**A**, **E**) PTP1B, (**B**, **F**) SHP-1, (**C**, **G**) SHP-2 and (**D**, **H**) YopH, relative to the distance between the center of mass of the WPD-loop and P-loop. All dRMSD values are calculated relative to the WPD-loop closed conformation of PTP1B (PDB IDs: 6B90^60^ and 3I80^61^ for (top) unliganded and (bottom) phosphoenzyme intermediate states, respectively). All simulations were initiated from the WPD-loop open conformation (in either (**A**-**D**) unliganded or (**E**-**G**) phosphoenzyme intermediate states). The corresponding data for the WPD-loop closed simulations is shown in Figure S3. Histograms were calculated based on 8 × 1.5 μs of sampling for each system in each conformational state (each panel). The purple stars indicate the position of the WPD-loop in the closed starting crystal structure and the black stars indicate the position of the WPD-loop in the open starting crystal structure for each system.

Following from this, we turned our focus to the relative stability of the WPD-loop of each enzyme of interest in the two conformational states (WPD-loop open/closed in either the unliganded or phosphoenzyme intermediate forms) of each enzyme, now also including both PTP1B and YopH in our analysis. To evaluate this, we constructed 2D-histograms of WPD-loop mobility as a function of distance root mean square deviations (dRMSD, Å) of the WPD-loops in each set of simulations relative to the structure of the WPD-loop of PTP1B in its catalytically closed conformation, as well as the corresponding distances between the center of mass of the WPD-loop and P-loop for each system (Figures 2 and S3). The dRMSD was defined by the set of all combined C_α_-atom distance deviations between all WPD-loop and all P-loop residues, using the PTP1B closed conformation to define our reference distances (Table S1).

In simulations initiated from the open state, the largest conformational space in the dRMSD dimension is sampled by YopH, as is to be expected based on prior experimental and computational work that indicate this loop is highly flexible.^22, 23, 62, 63^ We notice that SHP-1 and SHP-2 sample notably different regions in conformational space, with SHP-2 favoring more open states and SHP-1 favoring more closed states. However, these differences are reduced in the phosphoenzyme intermediate states in simulations of both SHP-1 and SHP-2. In contrast, in simulations initiated from the intermediate state, we observe the WPD-loop sampling closed conformations even when starting from open WPD-loops in all systems, with this effect being most pronounced in SHP-1 and SHP-2 (PTP1B also samples a semi-closed conformation). Further, when initiating simulations from the closed WPD-loop, we observe some loop opening in all unliganded systems, most clearly in SHP-2, whereas the presence of the phosphoenzyme intermediate tends to keep the loop in a closed conformation on the timescale of our simulations (8 × 1.5 μs sampling time per system), in part due to stabilizing interactions with the P-loop arginine as observed in our prior PTP1B/YopH study.^23^ We note that even in the phosphoenzyme intermediate closed WPD-loop starting state, SHP-2 does show limited WPD-loop opening, indicating that the closed WPD-loop state is far less stable in SHP-2 than other PTPs in this study (Figure S3). These results further correlate with the fact that almost all crystallized structures of SHP-2 available in the Protein Data Bank^64^ as of February 2026 (71/72) contain only open WPD-loops.

**Figure 3.**
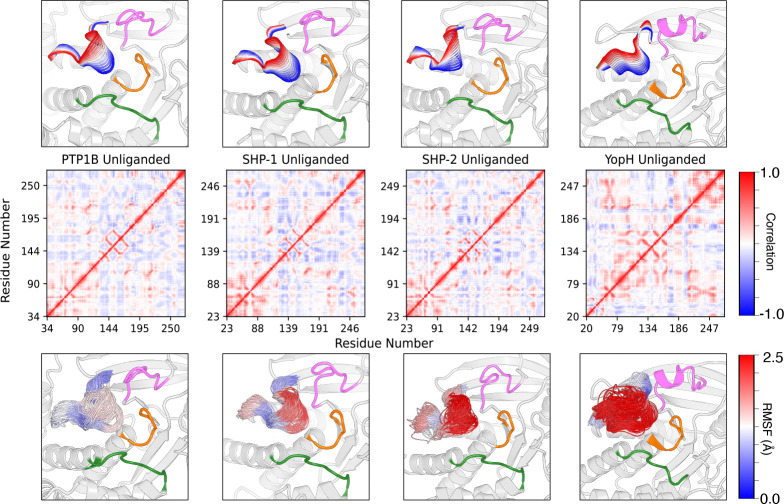
(**Center**) Dynamic cross-correlation maps (DCCM) calculated across the structurally conserved regions of the PTP1B, SHP-1, SHP-2 and YopH catalytic domains during MD simulations of each unliganded enzyme (initiated from both the open and closed WPD-loop conformational states). Analysis of the corresponding phosphoenzyme intermediate states is shown in Figure S4. Red indicates correlated and blue indicates anti-correlated motion. (**Top**) Projections along the dominant normal modes of movement of the WPD-loop for each system. (**Bottom**) Evenly spaced snapshots taken across all concatenated trajectories for each system, colored by RMSF of each residue. The Q-loop is colored in green, the P-loop in orange, and the E-loop in magenta. Note importantly that since this DCCM plot only shows structurally conserved regions that are common to all four PTPs for clarity, the edges of the plot are not the protein termini.

Furthermore, we have calculated dynamic-cross correlation maps (DCCM) based on our MD simulations (Figures 3 and S4). From this analysis, we observe a striking trend: in both unliganded and phosphoenzyme intermediate state simulations, the overall percentage of highly correlated residues tends to qualitatively increase with increasing WPD-loop flexibility, as shown by the conformational space sampled by each WPD-loop. This is interesting, as it is also counterintuitive – one would not immediately expect the scaffold to get more ordered as a mobile region becomes more flexible. We also observe that PTP1B, SHP-1, and SHP-2 exhibit similar properties whereby the flexibilities of WPD-loop residues are (comparatively) high close to the center and C-terminal edge of the loop, whereas YopH exhibits much higher flexibility throughout, allowing for a distinctive flexibility and opening predominantly around one side of the loop. This quality facilitates the characteristic ‘hyper-open’ states associated with YopH.^20^ Finally, as is to be expected, in all calculated DCCMs, we observe strong correlations on the diagonal, and increasingly strong correlations emanating from the diagonal (increasing with WPD-loop flexibility), but we also observe increasingly strong off-diagonal correlations with increasing WPD-loop flexibility. These include correlations between various key catalytic loops (WPD-, P-, Q- and E-loops), including increasingly strong correlations between the WPD- and E-loops (although in YopH these motions become anti-correlated). This latter observation is in good agreement with prior experimental and computational work, which emphasized the importance of coordinated motions of the WPD- and E-loops in HePTP, PTP1B and YopH.^23, 54, 65^

We additionally identify a region of correlated motion in the top left (bottom right) corner of each DCCM plot (Figures 3 and S4) that becomes increasingly prominent with increasing WPD-loop flexibility, moving from PTP1B through the SHPs to YopH. This region (Figure S5) corresponds to correlation between the motion of the α4- and α5-helices (using PTP1B numbering,^66^ or corresponding helices when not using PTP1B helix numbering), which is connected to the P-loop, and a nearby β-sheet (Figure S5). Note importantly that since this DCCM plot only shows structurally conserved regions that are common to all four PTPs for clarity, the edges of the plot are not the protein termini.

We further bring attention towards an interesting trend involving the correlated motion of the α3-helix (and corresponding helices), which is connected to the WPD-loop (Figure S5B). We notice an increase in anti-correlated motion between this helix and isolated regions interspersed among residues 124–174 on β-sheets β9 and β10 (PTP1B numbering, based on ref. ^67^) in PTP1B, SHP-1, and SHP-2, but then see an immediate shift to correlation in YopH, suggesting a much more complex relationship based on overall sequence of the system. This qualitative correlation with WPD-loop flexibility is particularly interesting given that in PTP1B, the α3-helix is part of an allosteric binding site that can be targeted to impair WPD-loop motion.^25, 51^ Further, incorporation of the C-terminus of the WPD-loop into the corresponding helix (α4) in YopH has been shown to allow the YopH WPD-loop to take on unusual hyper-open conformations, in both wild-type and chimeric forms of YopH,^20, 23^ and motion of the corresponding α3-helix has been shown to be correlated to WPD-loop motion in molecular simulation of YopH.^68^

### Impact of the SHP-1 and SHP-2 Tandem SH2 Domains on WPD-Loop Dynamics

As shown in Figures 1 and S1, SHP-1 and SHP-2 both carry tandem SH2 domains, and are in fact the only cytosolic PTPs to do so.^41^ Both proteins exhibit an autoinhibition mechanism whereby in the catalytically active ‘open’ state, the PTP domain is free to bind to substrate, while in the catalytically inactive ‘closed’ state, the active site is effectively blocked by the SH2 domains (Figure S1). Despite the similar mechanisms of autoinhibition of SHP-1 and SHP-2,^28, 29, 42–44^ however, the SH2 domains exert orthogonal effects on enzyme kinetics. Specifically, comparisons of the activities of full length and truncated (catalytic domain) versions of SHP-1 and SHP-2 have shown that in SHP-1, the presence of the SH2 domains primarily impacts *K*_M_ with *k*_cat_ largely unaffected,^30^ whereas in SHP-2, the *k*_cat_ of full-length SHP2 is reduced to 4.8% of the activity of the truncated catalytic domain.^45^ Given the importance of WPD-loop motion to catalysis and turnover in PTPs,^19, 22^ this suggests that the presence of the SH2 domains may have minimal impact on WPD-loop dynamics in SHP-1 (compared to the corresponding dynamics in only the truncated catalytic domain), whereas they likely have a substantive impact on WPD-loop dynamics in full length SHP2.

While there exist structures of SHP-1 in both catalytically active and autoinhibited conformations of the SH2 domains (PDB IDs: 3PS5^28^ and 2B3O,^29^ respectively), and of SHP-2 in its autoinhibited conformation (PDB ID: 4DGP^44^), in all these cases, the WPD-loop is in an open conformation. Therefore, there exists no structure of a fully activated SHP-1 or SHP-2 containing both open (non-autoinhibited) SH2 domains and a closed WPD-loop. Because of this, we must limit our comparison of full-length and truncated catalytic domain forms of SHP-1 and SHP-2 to the WPD-loop open conformation of the PTPs. Such analysis is still valuable however, as it has been previously shown that PTPs sample a range of interconverting loop-open conformations,^23, 54, 69^ and it has been suggested that there is a link between increased PTPase activity and increased flexibility of the open conformation of the WPD-loop.^21^ Therefore, simulations of the dynamics of the WPD-loop open state can still provide important insight into function.

**Figure 4.**
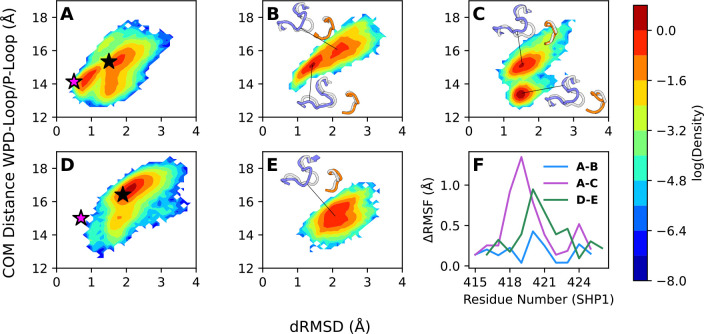
(**A**-**E**) 2D histograms of the distance root mean square deviations (dRMSD, Å, using WPD-loop-closed PTP1B as a reference) of the distances between the C_α_-atoms of all WPD-loop and all P-loop residues in comparison with the distance between the center of mass of the WPD and P-loops for (**A**-**C**) SHP-1 and (**D**, **E**) SHP-2, in the (**A**, **D**) catalytic domain only, (**B**, **E**) open WPD-loop and SH2-domains closed, and (**C**) open WPD-loop and SH2-domains open conformations. Histograms were calculated based on (**B**, **C**, **E**) 5 × 1 μs of sampling for each SH2-included system and (**A**, **D**) 8 × 1.5μs of sampling for the catalytic domain. Black stars indicate crystal structures of WPD-loop open catalytic domain, and magenta stars indicate crystal structures of WPD-loop closed catalytic domain. Projections are shown as trajectory snapshots associated with notable peaks with the WPD-loop shown in blue and P-loop shown in orange; aligned PTP1B closed unliganded structure is shown in gray as reference. (**F**) Comparison of root mean squared fluctuation (ΔRMSF) calculations for each residue of the WPD-loop between the catalytic domain only and full SH2 SHP-1 and SHP-2 structures; a positive value indicates more residue flexibility in the isolated catalytic domain. Legend notation corresponds with the histogram panels (*e.g.*, A-B indicates the calculated ΔRMSF of SHP1 catalytic domain – SHP1 autoinhibited structure).

To characterize the dynamics of the WPD-loop open state in these enzymes, we again generated 2D-histograms of WPD-loop mobility as a function of the dRMSD (Å) of the WPD-loops in each set of simulations relative to the structure of the WPD-loop of PTP1B in its catalytically closed conformation, and the distance (Å) between the centers of mass of the WPD- and P-loops (Figure 4). Our simulations indicate that, the presence of the SH2 domains of SHP-1 and SHP-2 alter the conformational space sampled by the WPD-loop, both with respect to the dynamics of the loop in the truncated catalytic domain, and between the two states of the SH2 domains (Figure 4).

In the case of both SHP-1 and SHP-2, structural data indicates that in the autoinhibited states, the Nβ4-Νβ5 loop of the N-SH2 domain protrudes into the catalytic domain, blocking the entrance to the active site (Figure S1).^29^ Further, an aspartic acid side chain on this loop enters into the active site, likely blocking complete WPD-loop closure through electrostatic repulsion between this residue and the catalytic aspartic acid on the WPD-loop (Figure S1). This would be expected to modify WPD-loop dynamics, as observed in our simulations (Figure 4). Although both SHP-1 and SHP-2 are autoinhibited in this same manner, we notice that with the addition of this interaction, the SHP-1 WPD-loop is able to sample both open and semi-closed WPD-loop conformations, while SHP-2 can only sample wide open conformations, in agreement with the observation that full-length SHP-1 and the truncated catalytic domain show similar turnover numbers.^30^ (Figure 4). In absence of this interaction, we also notice that SHP-1 can sample more semi-closed conformations, but also, interestingly, appears to undergo phosphate binding loop (P-loop motion) towards the active site (Figure 4). This is noteworthy, as while other classical PTPs typically possess rigid and structurally conserved P-loops, archaeal PTPs can undergo complex P-loop motions linked to active and inactive enzyme states, suggesting this P-loop motion may impact catalytic properties.^70–72^ Indeed, this unusual P-loop motion observed in SHP-1 likely contributes to the experimentally-observed SH2-mediated effect on *K*_M_ in this PTP, whereby the addition of the added SH2-domains alters substrate binding via subtle changes in active site loop conformations, while still allowing for proper WPD-loop motion, thus not altering *k*_cat_.^30^

In contrast, in simulations of both truncated catalytic domain and full-length autoinhibited SHP-2, we observe sampling of substantively more open WPD-loop conformations relative to SHP-1 (Figure 4), suggesting that the SH2 domains plausibly hamper effective WPD-loop closure to a greater degree in SHP-2 versus SHP-1, in agreement with significant loss of activity in full-length compared to truncated SHP-2.^45^ Our catalytic domain only simulations revealed that the SHP-2 closed WPD-loop state appears to be far less stable than that of SHP-1, despite high sequence similarities (Figure 2 and 4). Therefore, the tendency of SHP-2 to remain in a more open WPD-loop state compared to SHP-1 in the autoinhibited state could either be a result of differential allosteric impacts from the added SH2 domains between SHP-1 and SHP-2, or could simply be a result of the differences in dynamics that we observe for the catalytic domain only of these PTPs. Since in all cases, the added SH2 domains block the active site, preventing full WPD-loop closure, we chose to investigate how the flexibilities of WPD-loop residues are altered with the addition of the SH2-domains, to provide an indication as to whether the SH2 domains alter WPD-loop motion by simply blocking the active site or instead by altering loop mobility more broadly. To understand this further, we computed difference root mean squared fluctuations (ΔRMSF) for each of the WPD-loop residues between the isolated catalytic domain and the full SH2 structures to compare the impact of the added SH2 domains on WPD-loop flexibility and motion in both SHP-1 and SHP-2 (Figure 4F).

We notice that there is a fairly subtle but noticeable impact where the SH2 domains reduce the flexibility of the SHP-2 WPD-loop at the autoinhibited conformation and the SHP-1 WPD-loop at the SH2-open conformation. In particular, we notice greater flexibility in the H420 residue (SHP-1 numbering) for the SHP-2 catalytic domain only as compared to the autoinhibited structure and greater flexibility in the general acid D421 (SHP-1 numbering) for the SHP-1 catalytic domain only as compared to the SH2-open structure. The SH2 domains do not seem to meaningfully impact the flexibility of the majority of the SHP-1 WPD-loop residues at the autoinhibited state, except very slightly for residue H420. These data suggest, therefore, that the SH2 domains in the autoinhibited conformation reduce the flexibility of the WPD-loop only in SHP-2, presumably through allosteric effects, further decreasing the probably of sampling a catalytically active closed WPD-loop conformation once the SH2 domains shift to the open conformation. In other words, although both the isolated catalytic domain and autoinhibited SHP-2 structures sample open WPD-loop conformations, the autoinhibited structures maintain a higher level of rigidity, meaning it is likely more difficult to transition into a closed or semi-closed conformation once the SH2 domains shift to the open conformation. This observation aligns well with our conclusion that the SHP-2 catalytic domain can shift between semi closed and open structures, albeit greatly preferring open conformations, while the SHP-2 autoinhibited structure cannot (Figures 2 and 4). By contrast, the SHP-1 WPD-loop mobility is not notably altered when in the autoinhibited state. The altered dynamics we observe in Figure 4 are also important as they suggest that the SH2 domains are important not just for regulating substrate access to the active site, but also for controlling WPD-loop dynamics. These observations are particularly interesting in light of the fact that SHP-1 and SHP-2 have different biological functions *in vivo*.^41^

### Oncogenic mutations modulate active site loop dynamics via altered allosteric networks

There are an increasing range of methods with which to computationally characterize protein allostery.^73–75^ In prior work, we characterized allosteric communication pathways in both PTP1B and YopH using the shortest path map approach (SPM),^76^ demonstrating high conservation of allostery between these enzymes, with structure-based sequence alignment indicating that 35 of 69 SPM residues identified in PTP1B are conserved in YopH, despite only 17.5% sequence similarity between the two enzymes^23^ (note that this analysis was based on parallel-tempered metadynamics simulations;^77^ from our current conventional MD simulations, we observe 19/46 SPM residues being conserved in the two PTPs).

**Figure 5.**
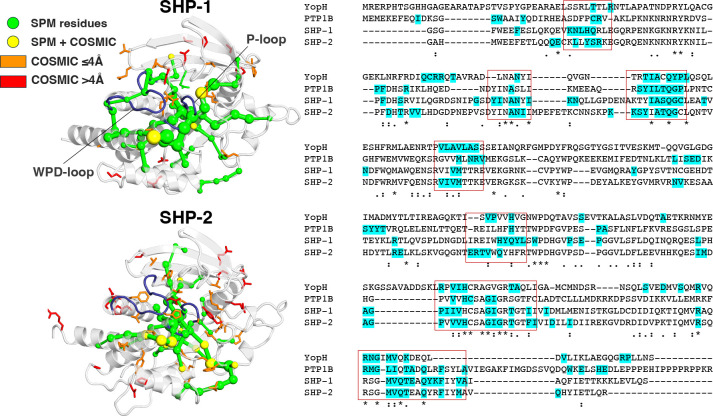
Identification of pathways utilized for allosteric communication in SHP-1 and SHP-2. **(left)** The shortest path map (SPM) obtained from our simulations of SHP-1 and SHP-2. Overlaid onto the graph are the residues with at least 2 known oncogenic mutations (obtained from the COSMIC database^59^) for SHP-1 and SHP-2. Mutations are in yellow if found on the SPM itself, orange if the closest atom distance to an SPM residue is <4 Å, and red the distance is ≥4Å. (**right**) Structure-based sequence alignment of PTP1B, SHP-1, SHP-2 and YopH, with SPM residues highlighted in blue. Boxes highlight regions with a high frequency of SPM residues in all four PTPs.

Given that PTP1B and SHP-1 have a higher 38% sequence similarity, and there is even higher sequence similarity between the SHP-1 and SHP-2 PTP domains (60%), one would expect even higher conservation of allostery between these enzymes (sequence similarity calculated using MUSCLE^55^). Indeed, projection of SPM residues onto structure-based sequence alignment of all four enzymes (Figure 5) suggests that, despite the differences in WPD-loop dynamics in these PTPs (Figure 2), >50% of SPM residues are conserved between SHP-1 and SHP-2 (32/61), and ~30–40% of SPM residues are conserved between pairs of SHP-1/SHP-2 and PTP1B/YopH (Table S2). Thus, there appears to be some degree of allosteric conservation across all four enzymes (which is highest among SHP-1 and SHP-2, which are an evolutionary pair^19^), in agreement with other work indicating conserved allostery across PTPs,^78, 79^ as well as our prior computational work on PTP1B and YopH.^23^

Following from this, and based on prior work that links oncogenic mutations to WPD-loop mutation in SHP-2,^71^ we compared the calculated allosteric pathways obtained by SPM analysis of SHP-1 and 2 with the locations of known oncogenic PTP-domain amino acid substitutions in the COSMIC database, as of 23^rd^ of February 2026.^59^ Based on COSMIC data, we identified 25 positions in the catalytic domain of SHP-1 and 41 positions in the catalytic domain of SHP-2 as being affected by two or more reported oncogenic amino acid substitutions. As can be seen from Figure 5 and Table S3, many (16/25 in SHP-1, and 35/50 in SHP-2) of the residues important to oncogenesis in these PTPs are directly or closely involved in an allosteric pathway. Based on this analysis, we selected the T501M and A323T substitutions in SHP-1, and N308D and Q506P substitutions in SHP-2 as variants of interest for further study, as they are both (1) located on the calculated SPM, and (2) known from either the literature or from the COSMIC database^59^ to be important to SHP-1/SHP-2 function (T501M and A323T in SHP-1 are both associated with adenocarinoma,^37, 59^ and N308D and Q506P in SHP-2 are additionally known to be gain-of-function Noonan/Leopard syndrome substitutions).^45, 80^. The positions of these amino acid substitutions relative to the SHP-1/SHP-2 active sites are shown in Figure S6.

**Figure 6.**
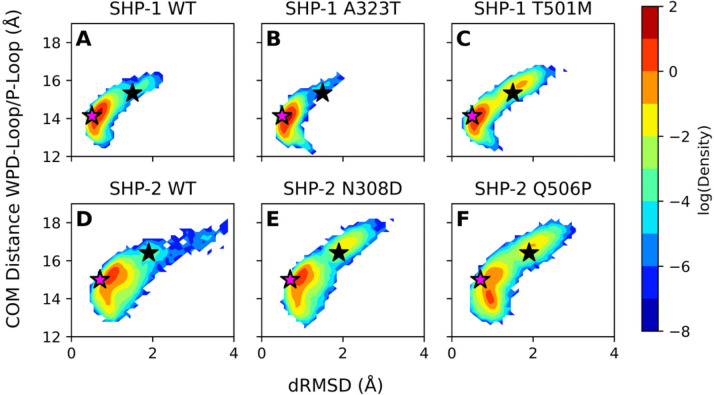
2D histograms of the distance root mean square deviations (dRMSD, Å) of the distances between the C_α_-atoms of all WPD-loop and all P-loop residues in (**A**) wild-type, (**B**) A323T, and (**C**) T501M SHP-1, and (**D**) wild-type, (**E**) N308D and (**F**) Q506P SHP-2, relative to the distance between the center of mass of the WPD-loop and P-loop. All dRMSD values are calculated relative to the WPD-loop closed conformation of PTP1B (PDB ID: 3I80^61^). All simulations were initiated from the WPD-loop closed conformation at the catalytically relevant phosphoenzyme intermediate start state. The corresponding data for the WPD-loop closed unliganded, open intermediate and open unliganded states are shown in Figures S7–S9. Histograms were calculated based on 8 × 1.5 μs of sampling for each system in each conformational state (each panel). The purple stars indicate the position of the WPD-loop in the closed starting crystal structure and the black stars indicate the position of the WPD-loop in the open starting crystal structure for each system.

For each variant of interest, we have performed 8 × 1.5μs MD simulations of the truncated catalytic domain of each PTP, initiated from closed and open WPD-loop conformations at both the unliganded and phosphoenzyme intermediates, as in our prior simulations described above. For simplicity, we specifically simulate the catalytic-domain only, to be able to test for direct links between these substitution and WPD-loop motion, without the additional layer of the SH2-domain autoinhibition mechanism. We again generated 2D histograms of WPD-loop mobility as a function of the dRMSD (Å) of the WPD-loops in each set of simulations relative to the structure of the WPD-loop of PTP1B in its catalytically closed conformation, as well as the distance (Å) between the centers of mass of the WPD- and P-loops. These data indicate significant differences in WPD-loop mobility between the different wild-type and mutant PTPs (Figures 6 and S7 to S9).

In the case of SHP-1, in simulations initiated from the catalytically relevant WPD-loop closed intermediate state (Figure 6) as well as the corresponding unliganded conformation (Figure S7), the WPD-loop of the A323T variant shows a narrower distribution of conformational states compared to wild-type SHP-1, continually favoring a closed state. Interestingly, despite the seemingly greater preference for closed WPD-loop structures in the A323T variant, our phosphoenzyme intermediate state simulations of the A323T SHP-1 variant consistently sample fewer catalytically relevant active site conformations than wild-type SHP-1 or even the SHP-1 T501M variant (Figure S10). Thus, even though the WPD-loop is more often in a closed state, the side chains of the general acid (D421) and the Q-loop Q500 (SHP1 numbering) are nevertheless less frequently pointed favorably towards the active site. In contrast, we see significantly more WPD-loop opening in the T501M variant, which is likely due to the longer, bulky side chain associated with the M501 residue, which can create a steric block for WPD-loop closure. Despite this, the T501M variant samples a greater fraction of reactive conformations than the A323T variant, despite having a lower tendency towards WPD-loop closure overall (Figure S10, note that in all cases, reactive conformations are sampled less than 10% of simulation time). Unfortunately, there is no data in the literature for the biochemical activity of the T501M variant; however, one would expect the impaired WPD-loop closure to lead to a loss of SHP-1 activity.

Following from this, in SHP-1 simulations initiated from the WPD-loop open intermediate state (Figure S8) we observe similar WPD-loop dynamics between wild-type SHP-1 and both the A323T and T501M variants, albeit with the T501M variant showing a slight preference for the open WPD-loop conformation. Further, in the simulations initiated from the open unliganded WPD-loop (Figure S9), we observe a more stabilized closed WPD-loop structure in the A323T variant as compared to wild-type, while the T501M variant fails to consistently sample a closed conformation, greatly favoring the open WPD-loop state.

In the case of SHP-2, where kinetic data on the activity of the catalytic domain of SHP-2 does exist for both the N308D (~2-fold increase in *k*_cat_ compared to wild-type SHP-2), and Q506P (~10-fold decrease in *k*_cat_ compared to wild-type SHP-2) variants,^45, 81–83^ we again observe subtle effects on WPD-loop dynamics from introduction of these substitutions onto the PTP scaffold. In the case of the catalytically relevant WPD-loop closed phosphoenzyme intermediate state, wild-type SHP-2 preferentially samples a WPD-loop closed conformation with some sampling of open conformations, whereas the N308D and Q506P variants sample both closed and open conformations. In contrast, when initiating sampling of the intermediate from a WPD-loop open conformation (Figure S8), introduction of the N308D substitution pushes the conformational equilibrium towards semi-closed conformations of the loop compared to wild-type SHP-2, while the Q506P variant continues to sample primarily open WPD-loop conformations, with limited semi-closed and closed conformations compared to the wild-type structure, in qualitative agreement with the impact of these substitutions on *k*_cat_. Thus, in both sets of simulations, the substitutions can expand the conformational space available to the WPD-loop. These effects are pronounced at both the intermediate state and in the unliganded enzyme forms; in the unliganded forms of SHP-2, the Q506P variant consistently samples more open conformations as opposed to the wild-type structure, irrespective of whether simulations are initiated from a WPD-loop closed (Figure S7) or open (Figure S9) conformation. Thus, the impact of the substitutions on loop dynamics manifests at all starting conformations, confirming the complex impact of singular oncogenic point substitutions on broader catalytic properties.

Ιn summary, we show here that in both PTPs, all pathogenic variants considered in this work impact the dynamics of the WPD-loop, even though these substitutions are not on the WPD-loop itself, but rather on the allosteric pathways controlling WPD-loop motion. This is significant, as it shows that, more globally, designing allosteric inhibitors that focus on the catalytic domain in order to target WPD-loop motion and prevent WPD-loop closure has promise to be an effective strategy for inhibiting both SHP PTPs.

### Empirical Valence Bond Calculations

**Figure 7.**
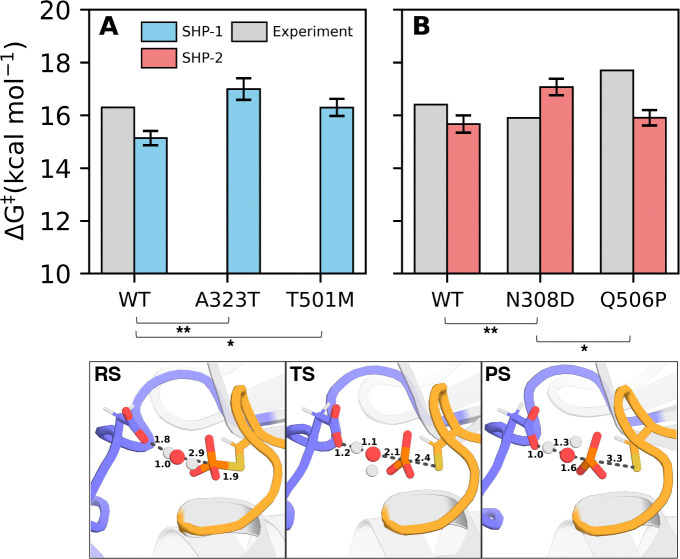
Comparison of experimental (gray) and calculated (blue/red) activation free energies (ΔG^‡^, kcal mol^−1^) for simulations of wild-type (WT) and variant forms of (**A**) SHP-1 and (**B**) SHP-2 initiated from structures extracted from molecular dynamics trajectories. Experimental values were derived from experimentally measured *k*_cat_ values at the conditions most relevant to our simulation setup, using transition state theory. The raw data for this figure, including the sources for the experimental data, are provided in Table S4. All calculated data are shown as average values and standard error of the mean over 30 independent empirical valence bond simulations. Stars indicate values which are statistically different (*p<0.05, **p<0.01) as calculated from a one-way ANOVA test followed by a Tukey Honestly Significant Difference (HSD) test.^86, 87^ Shown here are also representative reactant (RS), transition (TS) and product (PS) state structures from our EVB trajectories, extracted from the dominant cluster obtained from hierarchical agglomerative clustering on reacting heavy atoms during empirical valence bond (EVB)^84^ simulations of wild-type SHP-1, using CPPTRAJ.^88^ The equivalent structures for SHP-2 are shown in Figure S11, and the corresponding reactive distances are shown in Table S5.

From our molecular dynamics simulations, we have confirmed that the cancer-causing amino acid substitutions A323T and T501M in SHP-1 and N308D and Q506P in SHP-2 notably alter WPD-loop dynamics, contributing to their impacts towards catalysis in both these proteins. We supplemented this by performing empirical valence bond (EVB) simulations^84^ on these variants following the same protocol and parameters as in our prior PTP studies,^27, 54, 69, 85^ in order to determine whether the cancer-causing amino acid substitutions affect the rate-limiting hydrolysis step of the PTP mechanism. The resulting calculated reaction barriers are shown in Figure 7 and Table S4. Further, representative structures of key stationary points from EVB simulations of wild-type SHP-1 and SHP-1 are presented in Figures 7 and S11.

Based on these data, it can be seen that, in line with our prior EVB simulations of a range of PTPs including SHP-1,^23, 27, 54, 69^ our EVB calculations of wild-type SHP-1 and SHP-2 are in good agreement with the corresponding experimental values,^21, 30, 45, 56, 83, 89, 90^ especially given that it is plausible that WPD-loop motion is at least partially rate-limiting as in PTP1B.^22, 23^ In the case of SHP-1, we see an increase in activation free energy upon introduction of the A232T (p<0.01) and p<0.05) variants, in agreement with a reduction in reaction conformations upon disrupting the dynamics of the WPD-loop (Figure S10). This disruption is more pronounced in A232T than in T501M SHP-1, following the trend in activation free energies. In the case of SHP-2, we again observe an activation free energy increase in the N308D variant (p<0.01). We note that this contradicts kinetic measurements using the N308D SHP-2 catalytic domain, which suggest a slight *increase* in activity in this variant.^83^ However, more recent deep scanning mutagenesis data has suggested that mutations at position N308 in SHP-2 decrease intrinsic phosphatase activity, in line with our EVB calculations.^26^ Finally, we observe no notable difference in activation barrier between the SHP-2 wild-type and Q506P variant (p>0.05), indicating that experimentally observed loss of activity^45, 83^ is likely driven by altered loop dynamics rather than a chemical effect. Statistical significance was calculated using a one-way ANOVA test, followed by a Tukey Honestly Significant Difference (HSD) test.^86, 87^

Overall, these observations are in agreement with prior work,^27, 54, 69, 85^ where calculated activation energy differences between systems were small, as is to be expected given the nearly superimposable active sites of these enzymes. Taken together, these data indicate that as in our prior studies,^27, 54, 69, 85^ effects on the chemical steps can be negligible compared to the impact of loop motion on catalysis, due to the essential role of catalytic loop of PTPs in positioning a key catalytic residue in the PTP active site (Figure 1).

## Conclusions

The protein tyrosine phosphatases SHP-1 and SHP-2 are attractive but challenging drug targets for the treatment of cancer and a number of rare diseases,^10, 35, 37, 39, 40^ making them important topics of study. These enzymes have evolved as an evolutionary pair,^19^ and are the only known PTPs to carry tandem SH2 domains (Figure 1),^41^ which are involved in the autoinhibition of both SHP PTPs.^28, 29, 42–44^ Given the biomedical importance of these enzymes, the dynamics of SHP PTPs have been the subject of significant computational studies.^24, 91–105^ However, the majority of these have focused largely on the autoinhibition of these SHPs rather than the regulation of WPD-loop motion, despite the intimate link between loop dynamics and chemistry in PTPs.^22, 23^ Recent work, has, however, showcased that oncogenic mutations and allosteric drug inhibition impact WPD-loop motion in SHP-2.^26, 106^ We present here a detailed computational study of truncated catalytic domain and full length SHP-1 and SHP-2 (both wild-type enzymes and selected oncogenic variants), focusing on the impact of the SH2 domains and on oncogenic substitutions on WPD-loop dynamics and chemistry.

Importantly, we observe that the wild-type SHP-1 and SHP-2 proteins exhibit unique WPD-loop conformational ensembles despite their high sequence similarity, and these ensembles are also different to those exhibited by the more well-studied PTP1B and YopH PTPs. As SHP-1 and SHP-2 are enzymes that evolved as a pair through gene duplication,^19^ these clear differences in dynamics showcase how minor differences in sequence can allow for dramatically different function. Additionally, prior work has demonstrated a link between increased WPD-loop motion and higher PTPase activity.^21–23, 51, 107^ Here, our dynamic-cross correlation maps (Figures 3 and S4) demonstrate that motion of the WPD-loop is correlated not only with other functionally important loops such as the E- and Q-loops, but also key helices such as the α3-helix (PTP1B numbering), which has been implicated in both allosteric inhibition of PTP1B through blocking WPD-loop motion,^25, 51^ as well as the ability of YopH to assume hyper-open conformations of its WPD-loop.^68^ Counterintuitively, we also observe a qualitative link between increased correlated motions across the scaffold, and higher WPD-loop flexibility in both unliganded and phosphoenzyme intermediate state PTP simulations.

Following from this, we also observe significant differences in active site dynamics, both (1) between SHP-1 and SHP-2, (2) within each enzyme when starting from different chemical and conformational states (WPD-loop open/closed and unliganded *vs.* phosphoenzyme intermediate states) and (3) upon introduction of oncogenic substitutions. Importantly, as we and others have suggested,^23, 78, 79^ we demonstrate both conservation of allostery between SHP-1 and SHP-2, and, more significantly, the presence of several known hotspots for oncogenic mutations (based on the COSMIC database^59^) on the allosteric pathways that control WPD-loop motion in these enzymes (Figure 5).

Further characterization of selected SHP-1 and SHP-2 variants with substitutions at hotspot positions indicates that these substitutions do indeed alter WPD-loop dynamics, in ways that qualitatively align with the known or expected impact of these substitutions on catalysis (Figures 6, and S7 to S9), in agreement with prior work on SHP-2.^26^ Specifically, the effects of these mutations manifest themselves by primarily affecting protein dynamics (through modulating motion of the WPD-loop), which, in turn, impacts the chemical step of catalysis.

Finally, a comparison of WPD-loop dynamics in truncated (catalytic domain only) *vs.* full length (including SH2 domains) SHP-1 and SHP-2 shows that while the presence of the SH2 domains alters WPD-loop dynamics in both SHP-1 and SHP-2, how it actually does so differs between SHP1- and SHP-2 (Figure 4). Specifically, the SHP-2 SH2 domains lead to substantively more opening of the WPD-loop compared to in the truncated catalytic domain, whereas this effect is seen to a lesser extent in SHP-1. Further, we observe distortion of the phosphate binding P-loop in SHP-1, which is likely linked to impaired substrate binding. This qualitatively correlates with the different impact of the SH2 domains on catalysis in the two PTPs, including the much larger impact of autoinhibition on *k*_cat_ in SHP-2 than in SHP-1, whereas in SHP-1 the impact of the SH2 domains is primarily on *K*_M_ rather than *k*_cat_.^30, 45^

Taken together, our computational work provides detailed molecular insight into catalytic loop motion in these biomedically important PTPs and highlights key differences between the dynamical profiles of the two enzymes. Our data demonstrate that, while allosteric regulation of the autoinhibition mechanism is an attractive drug target in these enzymes (see *e.g.*, refs. ^108–113^, among others) WPD-loop motion is also important, and our data suggest that both SHP-1 and SHP-2 can likely be targeted by allosteric inhibitors focused on blocking WPD-loop closure, as has been achieved with other PTPs.^24–26^ More importantly, given that the impact of the SH2 domains on WPD-loop motion and catalysis differs between the two PTPs, this expands our repertoire of strategies to develop selective inhibitors for these critical but elusive anti-cancer drug targets.

## Materials and Methods

### Molecular Dynamics Simulations

Molecular dynamics simulations were performed using GROMACS^114^ version 2022.5 for all catalytic-domain only simulations, and the GPU-accelerated GROMACS version 2024.2 for all simulations of the full length SHP-1 and SHP-2 complexes including PTP-domains and SH2-domains. In all cases, the AMBER ff14SB^115^ forcefield was used along with the TIP3P water model,^116^ and simulation starting structures were generated using AmberTools22,^117^ utilizing the tLEaP and ParmEd libraries. A table of crystal structures used in this paper is shown in Table S6. For PTP catalytic domain only structures, simulations were performed in both the unliganded and phosphoenzyme intermediate forms of each protein, at both the WPD-loop closed and open conformations.

There exist a number of crystal structures of SHP-1 with alternate positions of the N-terminal helix (see PDB IDs 4HJP,^57^ 4GRZ^58^ and 8YHI^64^). In full-length SHP-1, this helix bridges the C-SH2 and PTP domains,^29^ and has been further suggested to play a cooperative role in substrate recognition.^118^ Since it was not clear which helix position was preferable for catalysis, we conducted benchmarks to determine the impact of helix position on WPD-loop motion. We used the Morph2 webserver (https://www.bioinformatics.org/pdbtools/morph2) to construct twelve SHP-1 starting structures, smoothly varying the starting position of the N-terminal helix for SHP-1 in both the closed WPD-loop state (PDB ID: 4GRZ^58^) and in the open WPD-loop state (PDB ID: 4HJP^57^) (24 simulations total), which we ran using the same equilibration procedure described below each followed by 500ns of production MD using GPU-accelerated GROMACS version 2024.2. Results indicated that helix positions located further from the main scaffold (such as those observed in the 4HJP^57^ and 8YHI^64^ structures) can be unstable and can affect WPD-loop motion *via* disrupted active site residue interactions (Figure S12). Based on this observation, we grafted the starting coordinates of the α_0_-helix in the 4GRZ^58^, which is closer to the protein scaffold (Figure S12), onto the 4HJP^57^ open-loop structure for our simulations. Parameters describing the phosphorylated cysteine along with all starting structures of PTP1B and YopH were taken from prior work.^23, 85^ For all oncogenic variants, amino acid substitutions were made in PyMOL^119^ using the Dunbrack rotamer library,^120^ and the best rotamers were chosen from visual inspection of possible clashes. Residues 313–315 in PDB ID: 4HJP^57^ (SHP-1 catalytic domain, WPD-loop open) were manually added using PDB ID: 4GRZ^58^ (SHP-1 in complex with PO_4_, WPD -loop closed) as a template.

The exists a SHP-2 structure (PDB 6CMQ^106^) containing models of both the open and closed WPD-loop conformations, and thus this structure was used for both SHP-2 open and closed starting structures. All other missing regions were fixed by using the Modeller^121^ extension of ChimeraX,^122^ generating 30 possible structures, and taking the structure with the lowest energy score for further simulations. In each unliganded PTP, the catalytic Asp and Cys residues (Figure 1) were kept in their deprotonated forms respectively. Protons were added to the structures using MolProbity,^123^ and PROPKA 3.1^124^ was utilized to identify any anomalous protonation states of ionizable residues, followed by extensive visual inspection. Based on this analysis, all other residues except the catalytic cysteine in unliganded PTPs were kept in their standard protonation states at physiological pH. We finally solvated each protein in a truncated octahedral water box extending 10Å away from the protein in all directions, and neutralized our systems with. Na^+^/Cl^−^ counterions.

Simulations were performed using the same protocol as described in prior work.^27^ In brief, the system was heated to 300K over 100ps using velocity rescaling,^125^ followed by 100 ps NPT production at 300K and 1 atm using a Parinello-Rahman barostat.^126^ Following this initial equilibration, we performed 100ns of further NPT equilibration before initiating 1.5 μs of production simulations in 8 replicas for each of wild-type SHP-1, SHP-2, oncogenic variants, PTP1B and YopH (catalytic domain only) and 5 replicas of 1μs for full-length SHP-1 and full-length SHP-2. All simulations of catalytic domain only PTPs were performed in each of the WPD-loop open/closed and both unliganded and phosphoenzyme intermediate states. This led to a total cumulative simulation time of 399 μs across all systems. Simulation convergence is shown in Figures S13 – S21. Additional simulation details are found in ref. ^27^.

Simulation analyses were carried out using CPPTRAJ for DCCM and normal mode analysis,^88^ and Plumed^127^ for dRMSD calculations, and MDAnalysis^128^ for all other analyses. We utilized the EMBL-EBI Job Dispatcher^129^ to conduct sequence alignments using MUSCLE,^55^ and used the Shortest Path Map method^76^ in order to compute allosteric pathways. All protein structures presented in this manuscript were visualized using PyMOL.^119^

### Empirical Valence Bond Simulations

We performed empirical valence bond (EVB)^84^ simulations of WT SHP-1, SHP-2, and oncogenic variants at the phosphoenzyme intermediate state, since the corresponding hydrolysis reaction is expected to be the rate-limiting step of this phosphatase-mediated reaction.^30–32^ All simulations were performed starting from the snapshots taken from our molecular dynamics simulations initiated at the WPD-closed conformation (catalytic domain only). Snapshots were considered productive if the Asp419(O)-pCys453(P) distance was less than 4.5Å and the Gln500(C)-pCys453(P) distance (SHP-1 numbering) was less than 8Å to ensure the Gln was able to point towards the active site and stabilize the catalytic water molecule (Figure S10). All system setup, equilibration and subsequent EVB simulations were performed as described in prior work,^27, 54, 69, 85^ using also the same EVB parameters as for prior studies. All EVB simulations were performed using the OPLS-AA force field.^130^ Each system was solvated in a 23 Å radius sphere of TIP3P water molecules^116^ centered on the phosphate atom of the phosphorylated cysteine, and all residues within the initial 85% of radius from the center of the sphere were ionized following the same procedure as for our standard molecular dynamics simulations. A list of ionization states used for our EVB simulations is presented in Table S7. This solvent sphere was described using the Surface Constrained All Atom Solvent Model (SCAAS),^131^ and long-range effects were described using the local reaction field approach.^132^ In each system, the nucleophilic water molecule as placed manually in the active site, aligned in an optimal position for catalysis relative to both nucleophilic attack on the phosphorus atom and deprotonation by the aspartic acid side chain. An identical procedure was used for all systems. Following 35 ns of initial equilibration, the convergence of which is shown in Figures S22 and S23, each EVB trajectory was propagated in 51 EVB mapping windows (*λ*_m_) of 200 ps in length each, with 30 independent EVB trajectories propagated per system. This resulted in 10.2 ns of EVB simulation time per trajectory, 306 ns per system, and 1.8 μs cumulative simulation time across all systems studied in this work.

## Supplementary Material

Supplement 1

Additional analysis of conformational flexibility, convergence plots, experimental and calculated activation free energies for EVB simulations, PDB structures used in this work, and ionization states of key residues in our EVB simulations are presented as Supporting Information. All raw data necessary to reproduce our MD and EVB simulations have been uploaded to Zenodo, DOI: 10.5281/zenodo.18817309.
