# Supplementary material for: Cancer-Causing Mutations Alter the Interplay Between Loop Dynamics and Catalysis in the Protein Tyrosine Phosphatases SHP-1 and SHP-2
Source: bioRxiv. 2026 Mar 3:2026.03.02.708844. Preprint. [Version 1] doi: 10.64898/2026.03.02.708844 (PMC13001400; doi:10.64898/2026.03.02.708844)
Supplement: Supplement 1 [file media-1.pdf]

Supporting Information for:

Cancer-Causing Mutations Alter the Interplay Between Loop Dynamics and Catalysis in the Protein Tyrosine Phosphatases SHP-1 and SHP-2

Alfie-Louise R. Brownless,[1] Michael Robinson,[2] and Shina C. L. Kamerlin[1,3,4*]

1. School of Chemistry and Biochemistry, Georgia Institute of Technology, 901 Atlantic Drive NW, Atlanta, Georgia 30332, USA
2. Department of Chemistry – BMC, Uppsala University, BMC Box 576, S-751 23 Uppsala, Sweden
3. School of Chemical and Biomolecular Engineering, Georgia Institute of Technology, 311 Ferst Drive, Atlanta, GA 30332, USA
4. Department of Chemistry, Lund University, Box 124, 221 00 Lund, Sweden

Corresponding author email address: skamerlin3@gatech.edu

Table of Contents

Supplementary Figures S3

Supplementary Tables S26

Supplementary References S37

Supplementary Figures

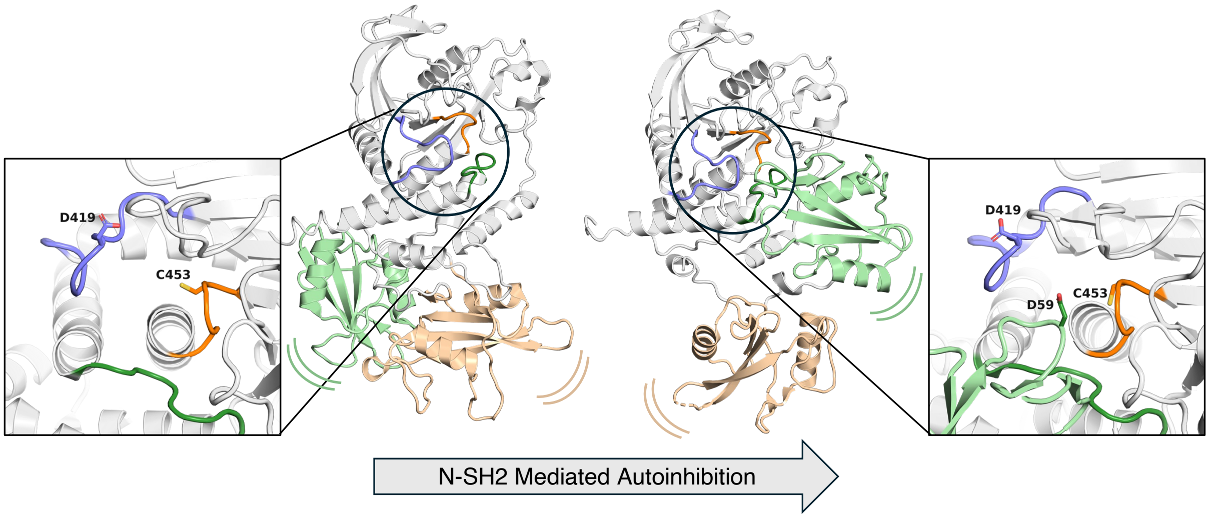

Figure S1. Visualization of N-SH2 domain autoinhibition, which is observed in both SHP-1 and SHP-2. In the non-autoinhibited state (left), the WPD-loop (blue) can move towards the P-loop (orange), resulting in a catalytically active conformation. By contrast, when autoinhibited (right), the N-SH2 domain (light green) interacts with the active site, preventing complete closure of the WPD-loop and thus preventing a catalytically active conformation from forming. The Q-loop is shown in dark green, and the C-SH2 domain is shown in tan. Note that structures shown are of SHP-1 in the open-SH2 state (PDB ID: 3PS5[1]), and in autoinhibited state (PDB ID: 2B3O[2]).

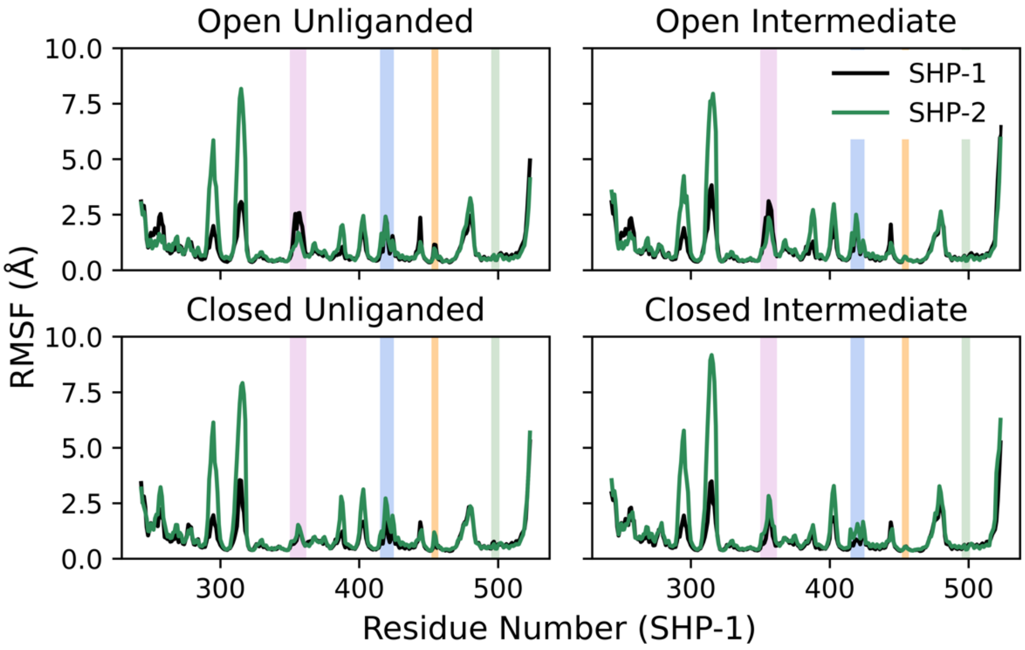

Figure S2. Root mean squared fluctuations (RMSF, Å) of all C_{α}-atoms of wild-type SHP-1 and SHP-2 calculated across 8 x 1.5μs concatenated MD trajectories. Regions of interest were aligned using MUSCLE.[3] Shading indicate key active site loop regions as shown in **Figure 1**: E-loop (pink), WPD-loop (blue), P-loop (orange), Q-loop (green).

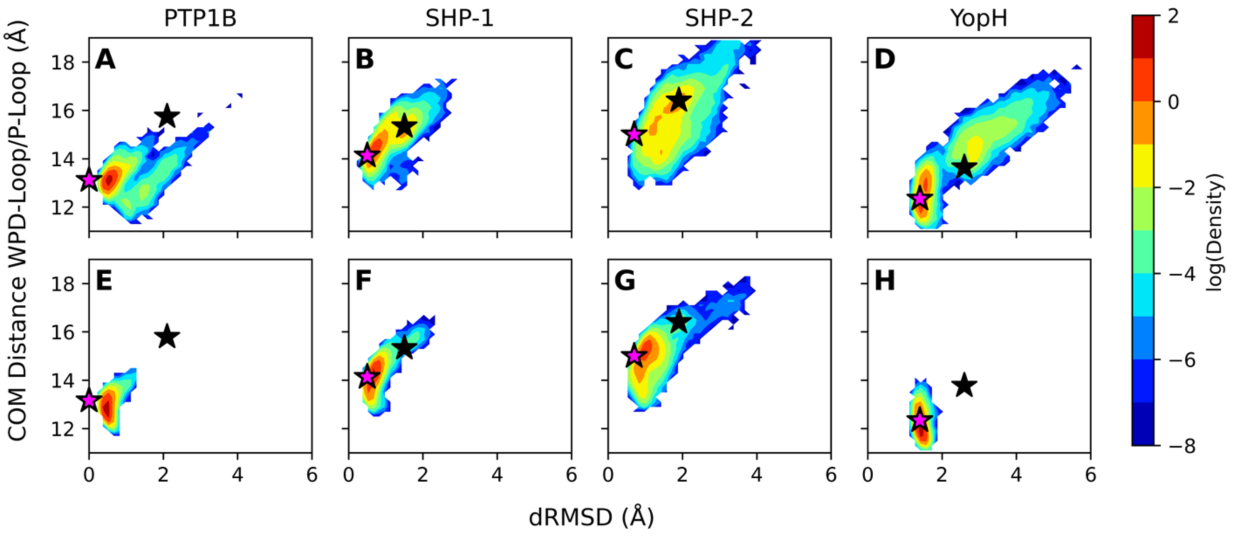

Figure S3. 2D histograms of the distance root mean square deviations (dRMSD, Å) of the distances between the C_α-atoms of all WPD-loop and all P-loop residues in wild-type, (**A**, **E**) PTP1B, (**B**, **F**) SHP-1, (**C**, **G**) SHP-2 and (**D**, **H**) YopH, relative to the distance between the center of mass of the WPD-loop and P-loop. All dRMSD are calculated relative to the WPD-loop closed conformation of PTP1B (PDB IDs: 6B90[4] and 3I80[5] for the (top) unliganded and (bottom) phosphoenzyme intermediate states, respectively). All simulations were initiated from the WPD-loop closed conformation (in either (**A-D**) unliganded or (**E-G**) phosphoenzyme intermediate states). The corresponding data for the WPD-loop open simulations is shown in **Figure 2**. Histograms were calculated based on 8 x 1.5 μs of sampling for each system in each conformational state (each panel). The purple stars indicate the position of the WPD-loop in the closed starting crystal structure and the black stars indicate the position of the WPD-loop in the open starting crystal structure for each system.

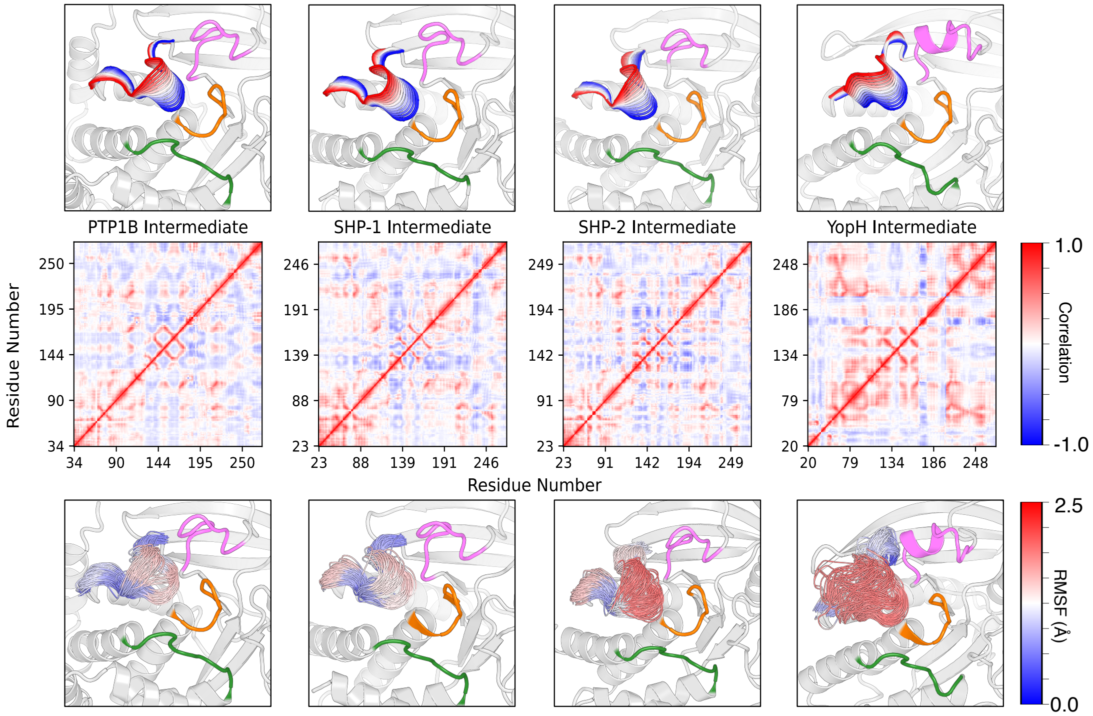

Figure S4. (**Center**) Dynamic cross-correlation maps (DCCM) calculated across the structurally conserved regions of the PTP1B, SHP-1, SHP-2 and YopH catalytic domains, during MD simulations of the phosphoenzyme intermediate (initiated from both the WPD-loop open and closed conformational states). Analysis of the corresponding unliganded states is shown in **Figure 3**. Red indicates correlated and blue indicates anti-correlated motion. (**Top**) Projections along the dominant normal modes of movement of the WPD-loop for each system. (**Bottom**) Evenly spaced snapshots taken across concatenated trajectories for each system, colored by the root mean square fluctuations (RMSF, Å) of each residue during our simulations. The Q-loop is colored in green, the P-loop in orange, and the E-loop in magenta.

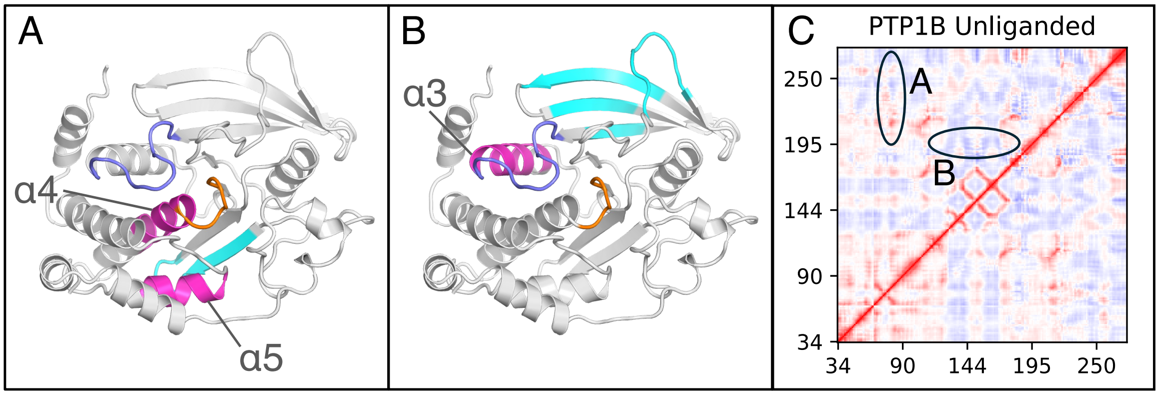

Figure S5. Projections of notable regions as identified from our correlation analysis (**Figure 3** of the main text), using PTP1B as a representative system to showcase regions of interest. Features in magenta correlate with regions in cyan, as discussed in the main text. Shown here are (**A**) Residues located on the the α4- and α5-helices (PTP1B helix numbering) which exhibit notable increases in correlation as WPD-loop flexibility increases (**Figure 3**). (**B**) The α3-helix (PTP1B helix numbering), which shows an increase in anti-correlation with the neighboring β-sheet as WPD-loop flexibility increases, with the exception of YopH which shows increased correlation in that region (**Figure 3**). (**C**) The calculated dynamic cross-correlation map (DCCM) for unliganded PTP1B, with regions of interest highlighted. In panels **A** and **B**, the WPD-loop is shown in blue, and the P-loop is shown in orange.

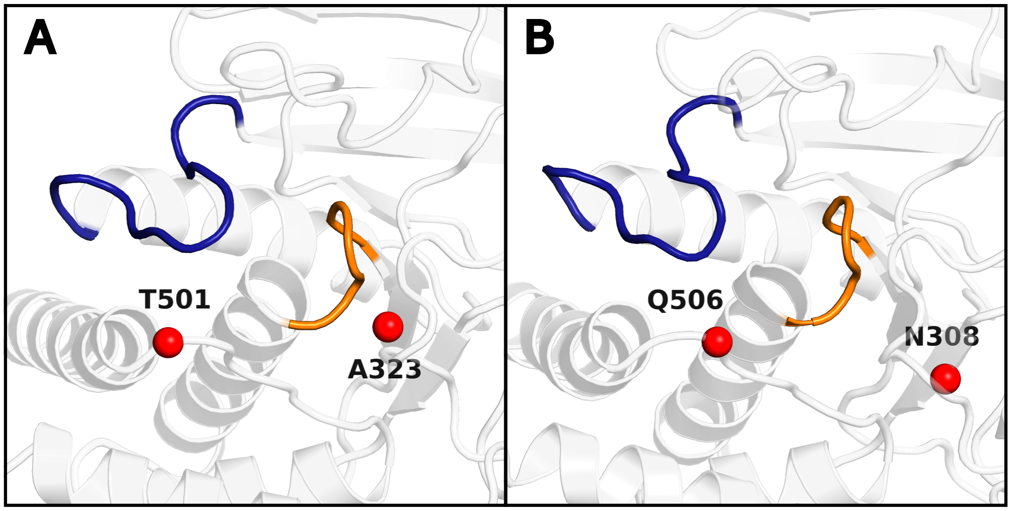

Figure S6. Location of the pathogenic amino acid substitutions studied in this work relative to the (**A**) SHP-1 and (**B**) SHP-2 active sites. The WPD-loop is shown in blue, and P-loop is shown in orange. The following SHP variants were considered: A323T, T501M (SHP-1), and Q506P, N308D (SHP-2).

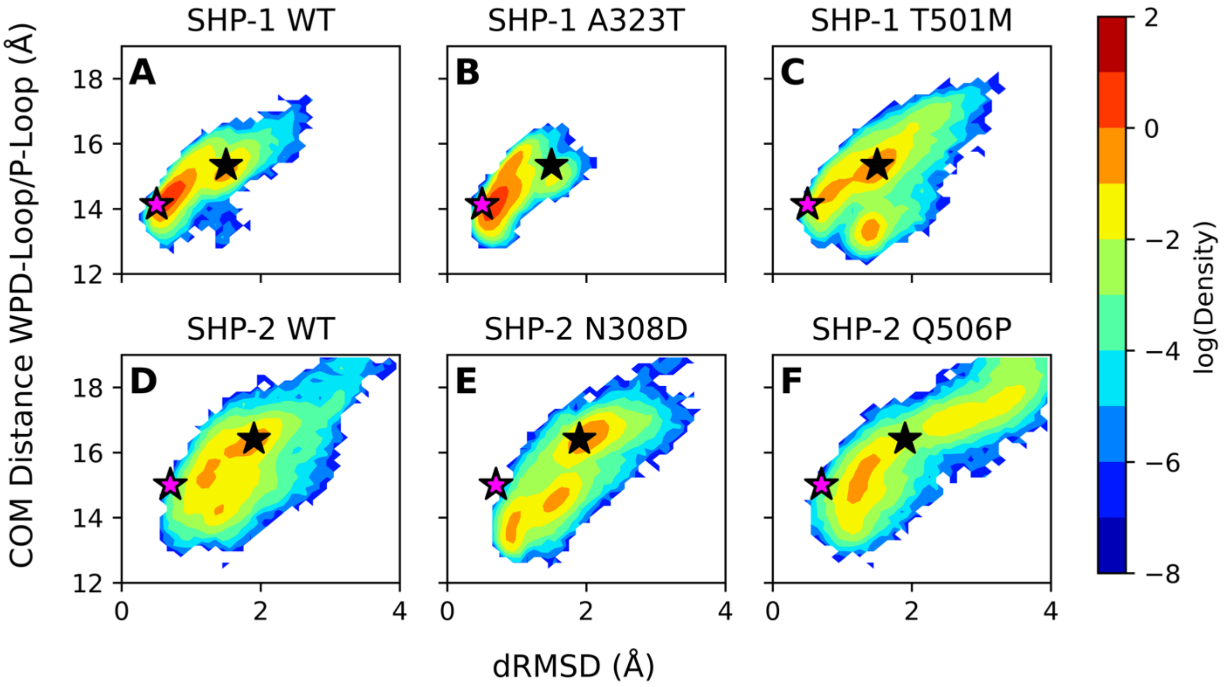

Figure S7. 2D histograms of the distance root mean square deviations (dRMSD, Å) of the distances between the C_α-atoms of all WPD-loop and all P-loop residues in (**A**) wild-type, (**B**) A323T, and (**C**) T501M SHP-1, and (**D**) wild-type, (**E**) N308D and (**F**) Q506P SHP-2, relative to the distance between the center of mass of the WPD-loop and P-loop. All dRMSD values are calculated relative to the WPD-loop closed conformation of PTP1B (PDB ID: 3I80[5]). All simulations were initiated from the WPD-loop closed conformation in the unliganded start state. The corresponding data for the WPD-loop closed intermediate, open intermediate, and open unliganded states are shown in **Figures 6, S8** and **S9**. Histograms were calculated based on 8 x 1.5 μs of sampling for each system in each conformational state (each panel). The purple stars indicate the position of the WPD-loop in the closed starting crystal structure and the black stars indicate the position of the WPD-loop in the open starting crystal structure for each system.

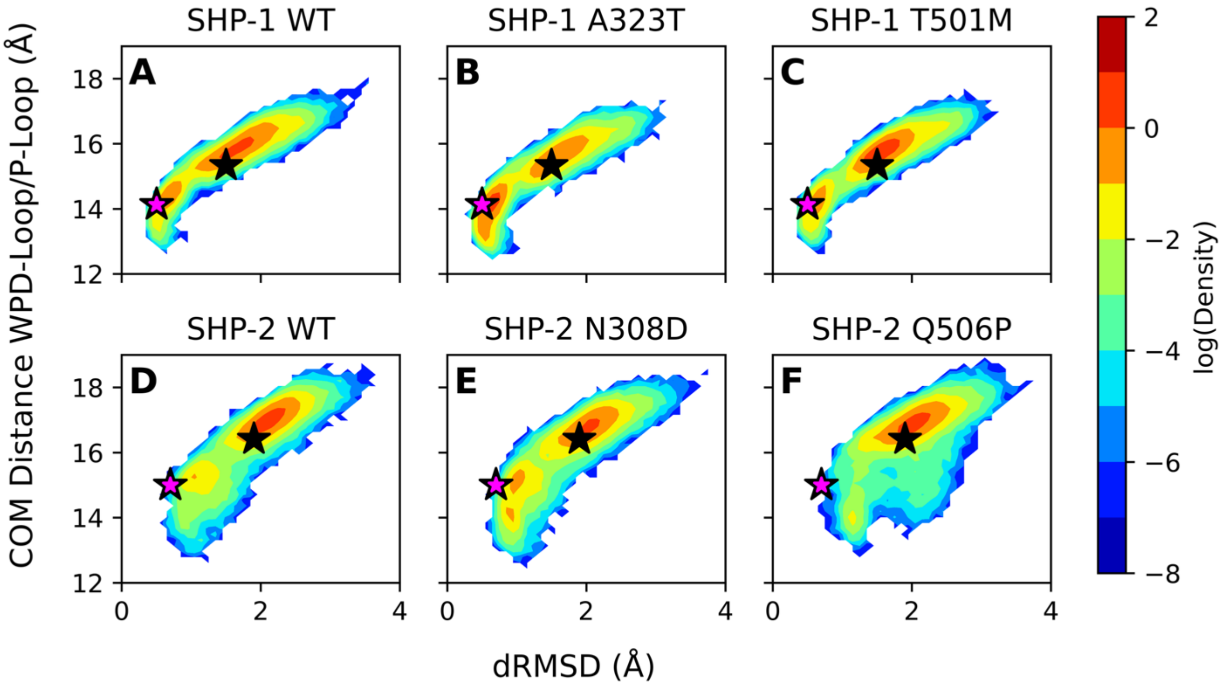

Figure S8. 2D histograms of the distance root mean square deviations (dRMSD, Å) of the distances between the C_α-atoms of all WPD-loop and all P-loop residues in (**A**) wild-type, (**B**) A323T, and (**C**) T501M SHP-1, and (**D**) wild-type, (**E**) N308D and (**F**) Q506P SHP-2, relative to the distance between the center of mass of the WPD-loop and P-loop. All dRMSD values are calculated relative to the WPD-loop closed conformation of PTP1B (PDB ID: 3I80[5]). All simulations were initiated from the WPD-loop open conformation in the phosphoenzyme intermediate start state. The corresponding data for the WPD-loop closed intermediate, closed unliganded, and open unliganded states are shown in **Figures 6, S7** and **S9**. Histograms were calculated based on 8 x 1.5 µs of sampling for each variant system in each conformational state (each panel). The purple stars indicate the position of the WPD-loop in the closed starting crystal structure and the black stars indicate the position of the WPD-loop in the open starting crystal structure for each system.

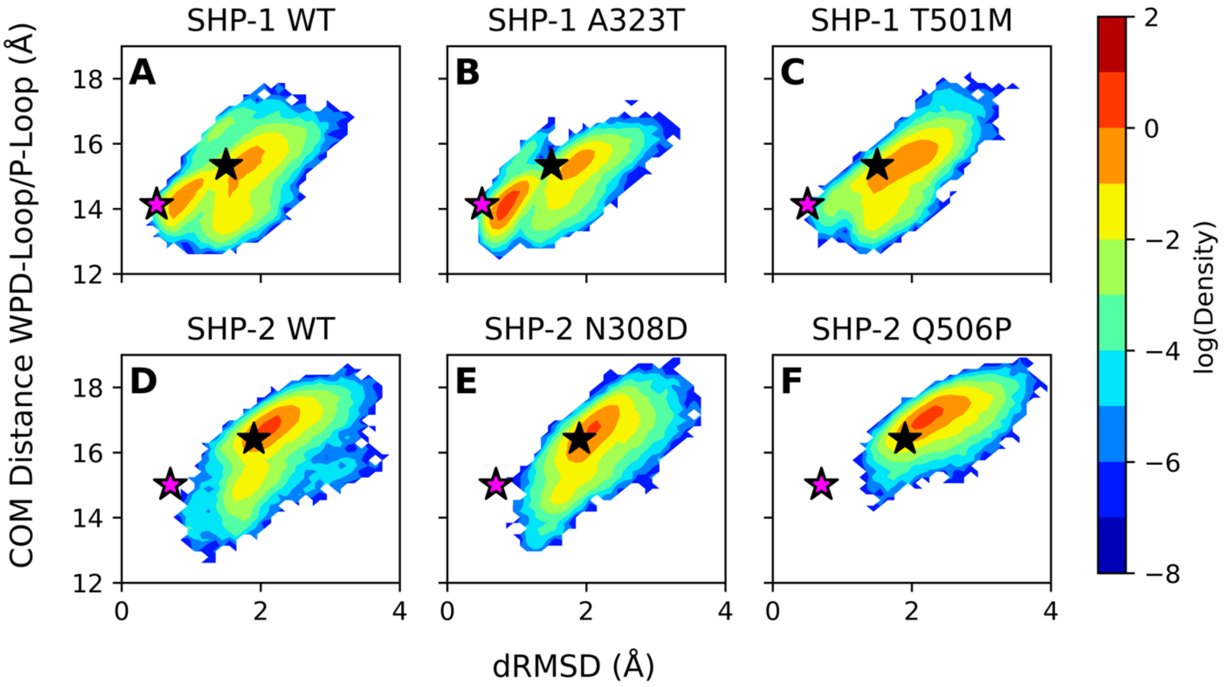

Figure S9. 2D histograms of the distance root mean square deviations (dRMSD, Å) of the distances between the C_α-atoms of all WPD-loop and all P-loop residues in (**A**) wild-type, (**B**) A323T, and (**C**) T501M SHP-1, and (**D**) wild-type, (**E**) N308D and (**F**) Q506P SHP-2, relative to the distance between the center of mass of the WPD-loop and P-loop. All dRMSD values are calculated relative to the WPD-loop closed conformation of PTP1B (PDB ID: 3I80[5]). All simulations were initiated from the WPD-loop open conformation in the unliganded start state. The corresponding data for the WPD-loop closed intermediate, closed unliganded, and open intermediate states are shown in **Figures 6, S7** and **S8**. Histograms were calculated based on 8 x 1.5 μs of sampling for each system in each conformational state (each panel). The purple stars indicate the position of the WPD-loop in the closed starting crystal structure and the black stars indicate the position of the WPD-loop in the open starting crystal structure for each system.

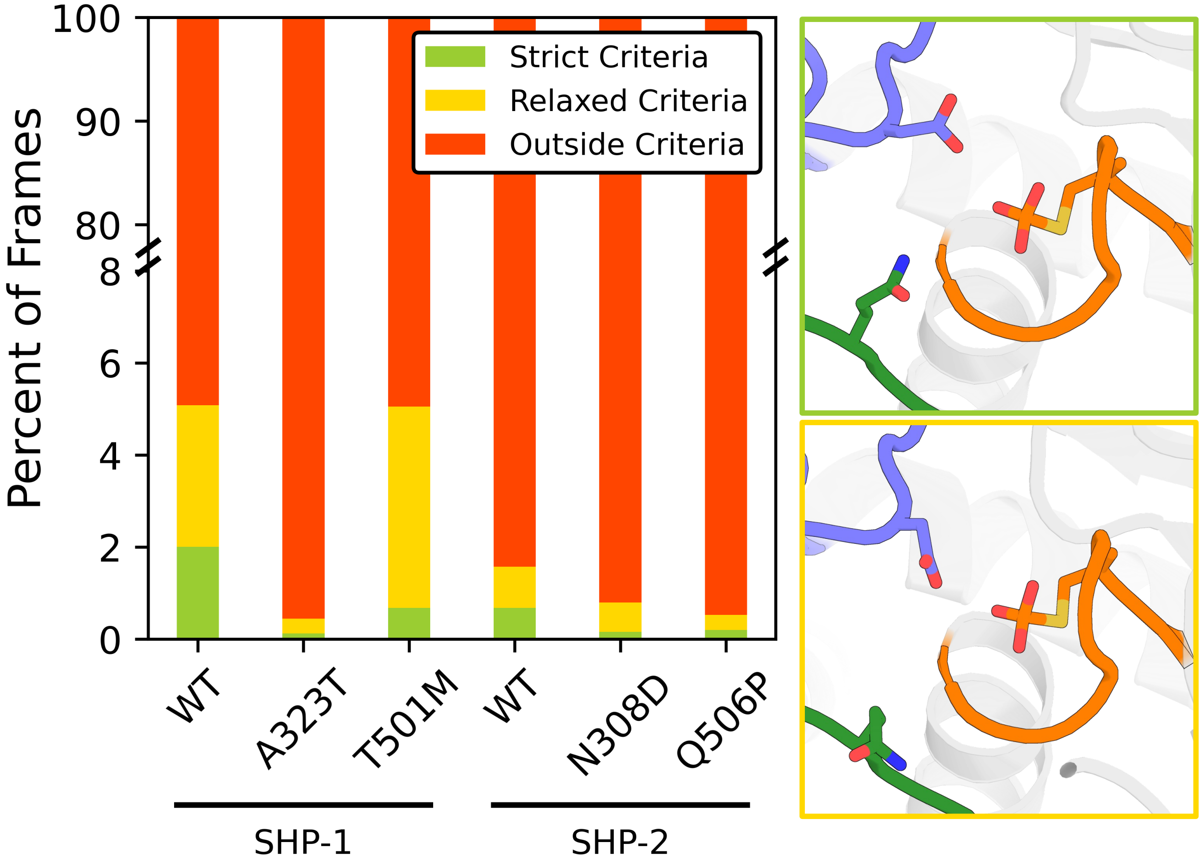

Figure S10. Percentage of molecular dynamics simulation frames at the closed WPD-loop phosphoenzyme intermediate starting state in which the active site is found in a reactive conformation for hydrolysis, based on distances between the catalytic Asp on the WPD-loop, the Gln on the Q-loop, and the phosphorus atom at the phosphoenzyme intermediate. Structures were classified as obeying "strict criteria" (top right) if the Asp419(O)-pCys453(P) distance was less than 4.5Å and the Gln500(C)-pCys453(P) distance (SHP-1 numbering) was less than 8Å, in order to ensure that the Gln side chain was able to point towards the active site and stabilize the catalytic water molecule. Structures were further classified as obeying "relaxed criteria" (bottom right) if the Asp419(O)-pCys453(P) distance was less than 4.8Å and the Gln500(C)-pCys453(P) distance was less than 12Å.

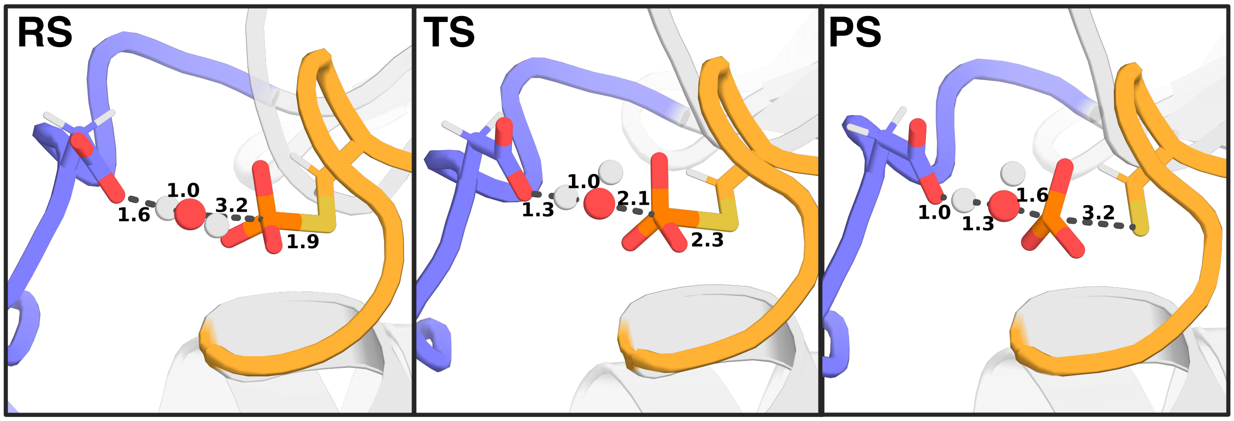

Figure S11. Shown here are also representative reactant (RS), transition (TS) and product (PS) state structures from our EVB trajectories, extracted from the dominant cluster obtained from hierarchical agglomerative clustering on reacting heavy atoms during empirical valence bond (EVB)[6] simulations of wild-type SHP-2, using CPPTRAJ.[7] The equivalent structures for SHP-2 are shown in **Figure 7**, and the corresponding reactive distances are shown in **Table S5**.

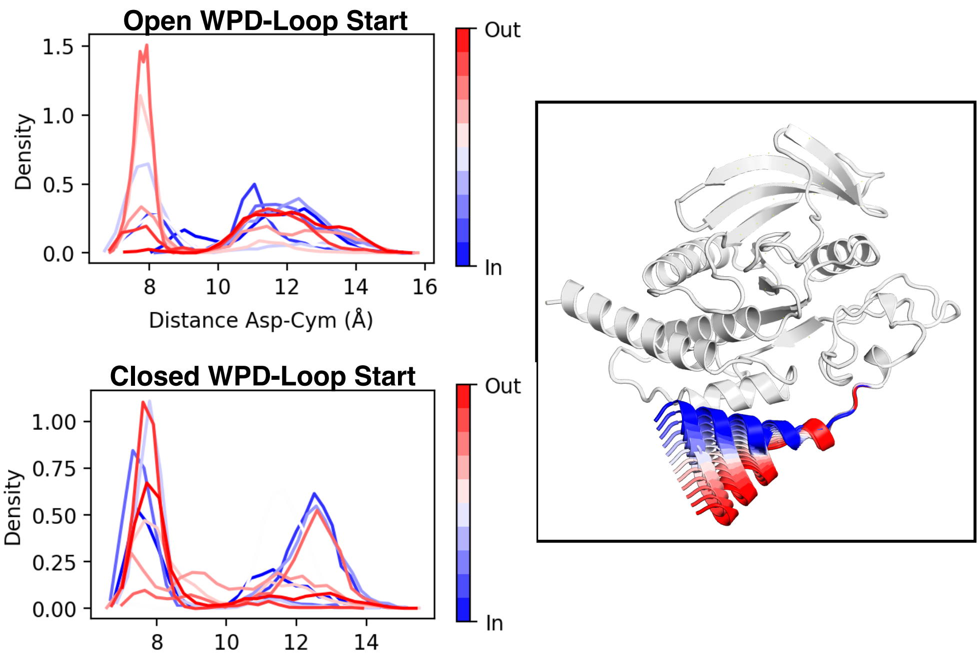

Figure S12. Benchmarking the effect of initializing N-terminal helix position on WPD-loop motion in SHP-1. Starting snapshots of helix positions were obtained using the Morph2 webserver (available at https://www.bioinformatics.org/pdbtools/morph2), using the helix position as defined from the PDB ID: 4GRZ[8] crystal structure as the 'in' conformation and the position as defined by the PDB ID: 4HJP[9] crystal structure as the 'out' conformation. 500ns simulations were run for each helix position seed.

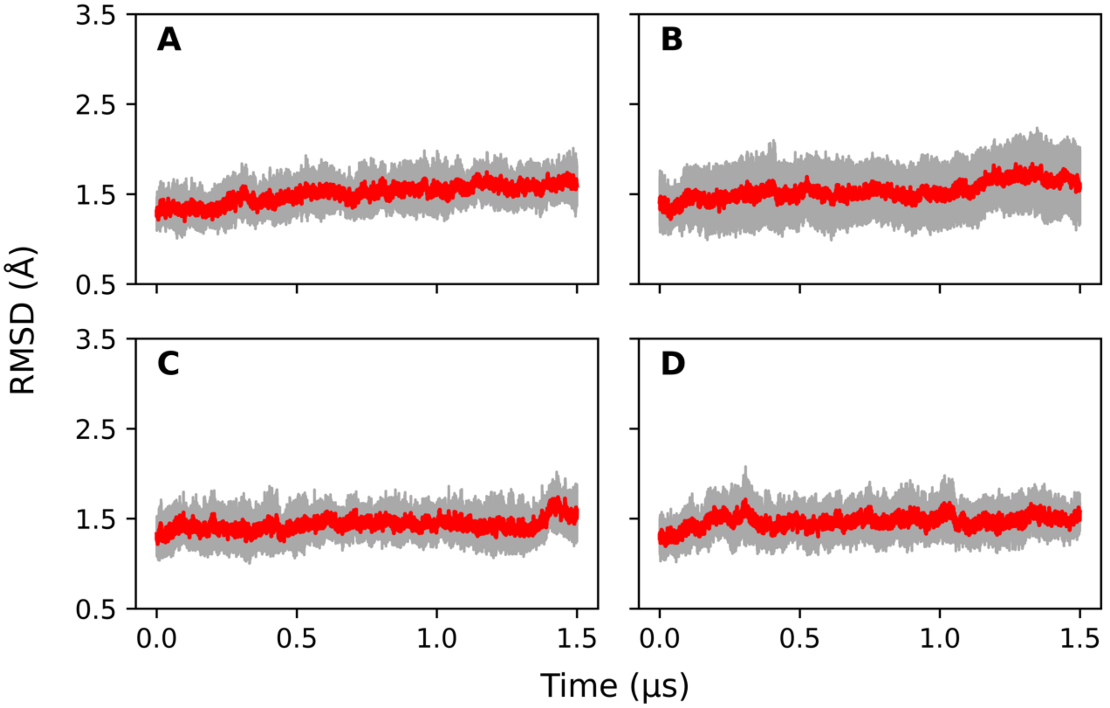

Figure S13. Root mean squared deviations (RMSD (Å)) calculated for all C_α-atoms of the catalytic domain of wild-type SHP-1, calculated from simulations initiated from the (**A**) WPD-loop open and unliganded, (**B**) WPD-loop open and phosphoenzyme intermediate, (**C**) WPD-loop closed and unliganded, and (**D**) WPD-loop closed and phosphoenzyme intermediate states. The red lines indicate averages, and gray shading indicates standard deviations calculated over 8 x 1.5μs independent MD replicas.

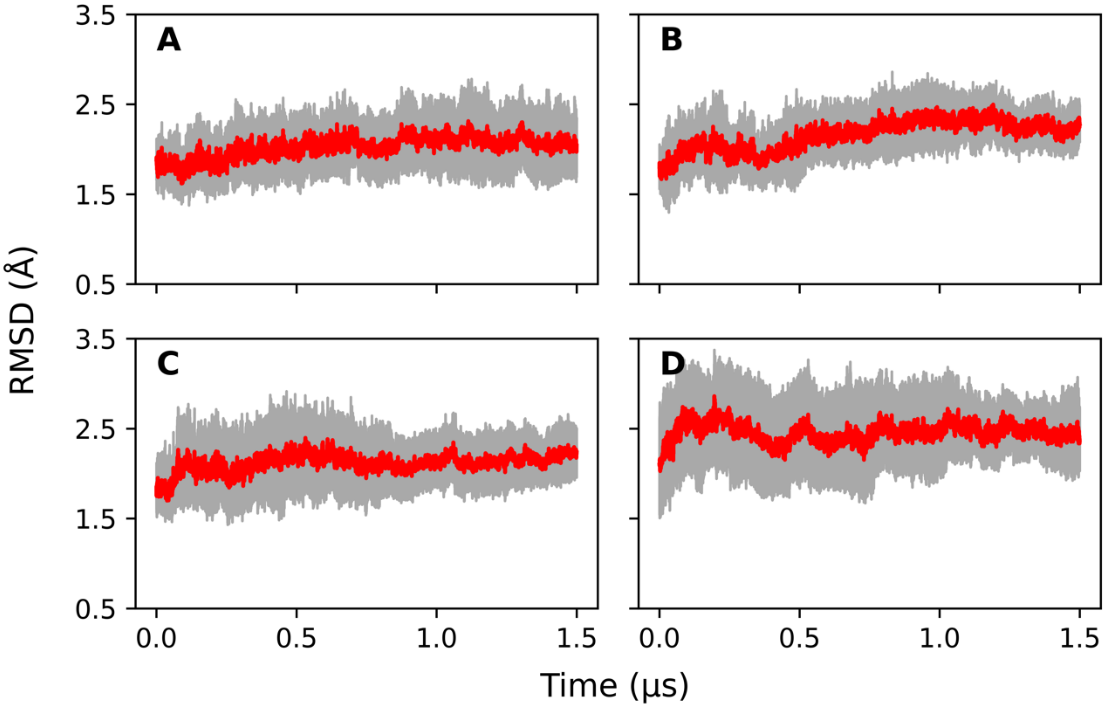

Figure S14. Root mean squared deviations (RMSD (Å)) calculated for all C_α-atoms of the catalytic domain of wild-type SHP-2, calculated from simulations initiated from the (**A**) WPD-loop open and unliganded, (**B**) WPD-loop open and phosphoenzyme intermediate, (**C**) WPD-loop closed and unliganded, and (**D**) WPD-loop closed and phosphoenzyme intermediate states. The red lines indicate averages, and gray shading indicates standard deviations calculated over 8 x 1.5μs independent MD replicas.

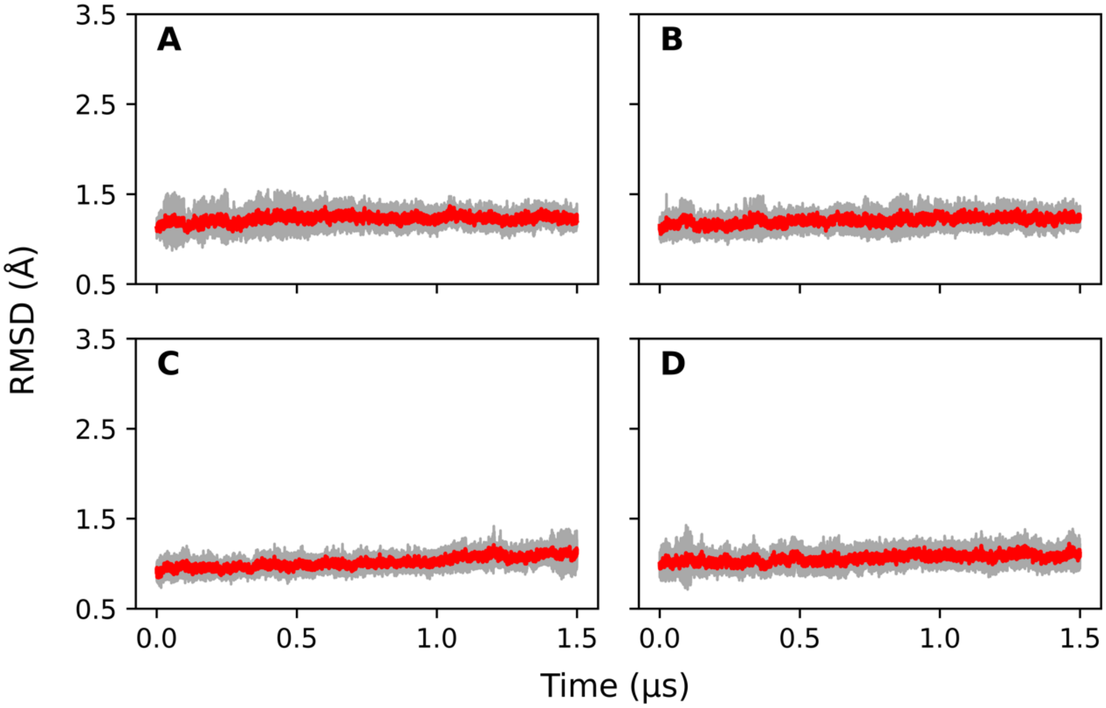

Figure S15. Root mean squared deviations (RMSD (Å)) calculated for all C_α-atoms of the catalytic domain of PTP1B, calculated from simulations initiated from the (**A**) WPD-loop open and unliganded, (**B**) WPD-loop open and phosphoenzyme intermediate, (**C**) WPD-loop closed and unliganded, and (**D**) WPD-loop closed and phosphoenzyme intermediate states. The red lines indicate averages, and gray shading indicates standard deviations calculated over 8 x 1.5μs independent MD replicas.

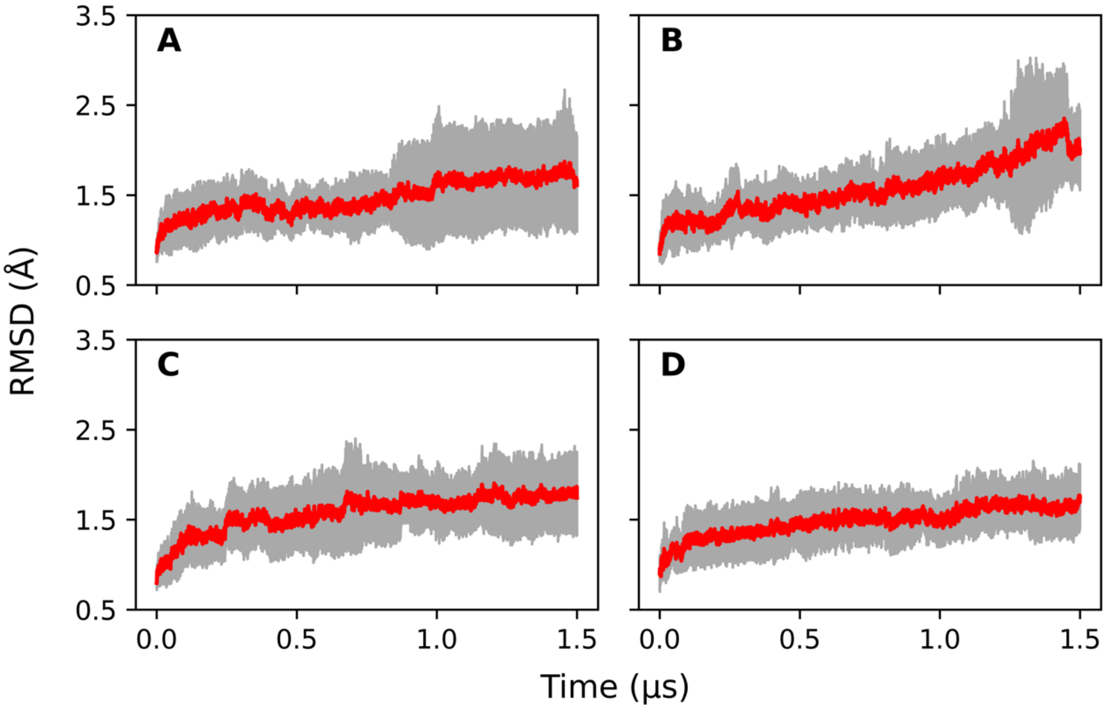

Figure S16. Root mean squared deviations (RMSD (Å)) calculated for all C_α-atoms of the catalytic domain of YopH, calculated from simulations initiated from the (**A**) WPD-loop open and unliganded, (**B**) WPD-loop open and phosphoenzyme intermediate, (**C**) WPD-loop closed and unliganded, and (**D**) WPD-loop closed and phosphoenzyme intermediate states. The red lines indicate averages, and gray shading indicates standard deviations calculated over 8 x 1.5μs independent MD replicas.

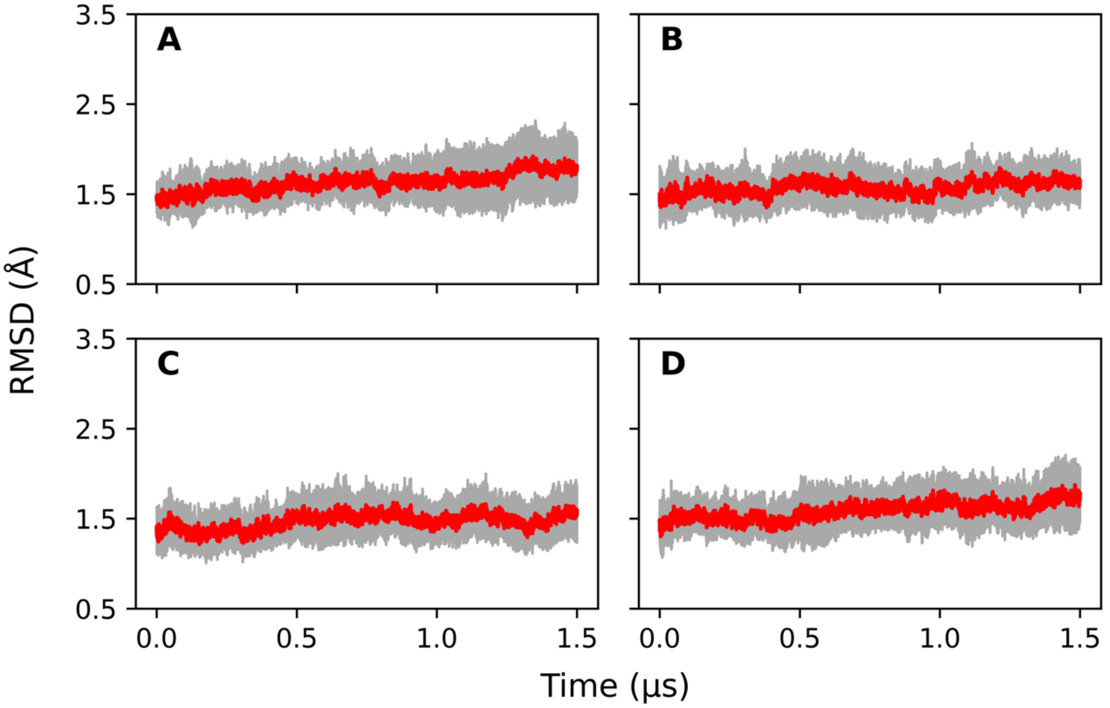

Figure S17. Root mean squared deviations (RMSD (Å)) calculated for all C_{α}-atoms of the catalytic domain of A323T SHP-1, calculated from simulations initiated from the (**A**) WPD-loop open and unliganded, (**B**) WPD-loop open and phosphoenzyme intermediate, (**C**) WPD-loop closed and unliganded, and (**D**) WPD-loop closed and phosphoenzyme intermediate states. The red lines indicate averages, and gray shading indicates standard deviations calculated over 8 x 1.5μs independent MD replicas.

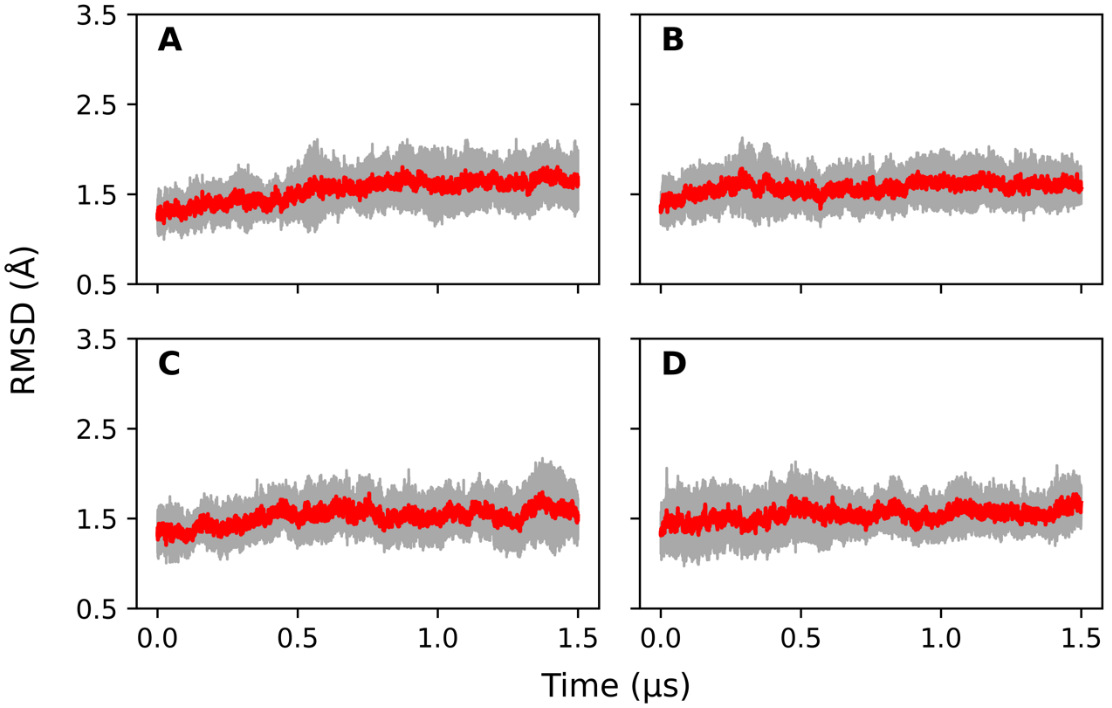

Figure S18. Root mean squared deviations (RMSD (Å)) calculated for all C_{α}-atoms of the catalytic domain of T501M SHP-1, calculated from simulations initiated from the (**A**) WPD-loop open and unliganded, (**B**) WPD-loop open and phosphoenzyme intermediate, (**C**) WPD-loop closed and unliganded, and (**D**) WPD-loop closed and phosphoenzyme intermediate states. The red lines indicate averages, and gray shading indicates standard deviations calculated over 8 x 1.5μs independent MD replicas.

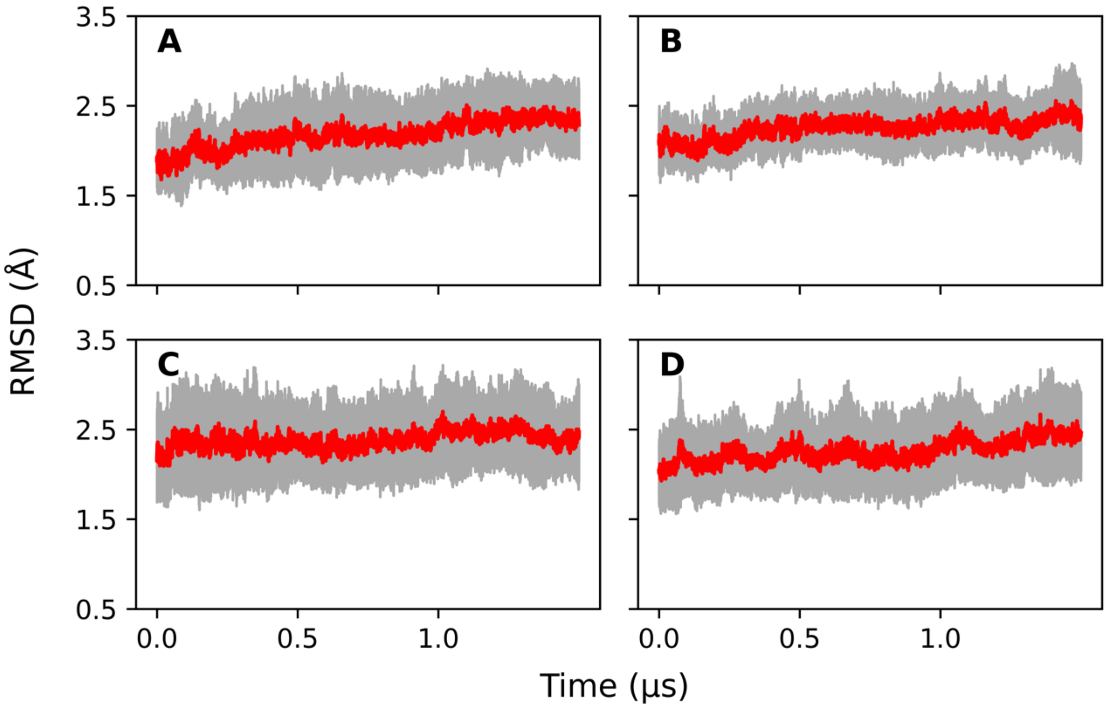

Figure S19. Root mean squared deviations (RMSD (Å)) calculated for all C_α-atoms of the catalytic domain of N308D SHP-2, calculated from simulations initiated from the (**A**) WPD-loop open and unliganded, (**B**) WPD-loop open and phosphoenzyme intermediate, (**C**) WPD-loop closed and unliganded, and (**D**) WPD-loop closed and phosphoenzyme intermediate states. The red lines indicate averages, and gray shading indicates standard deviations calculated over 8 x 1.5μs independent MD replicas.

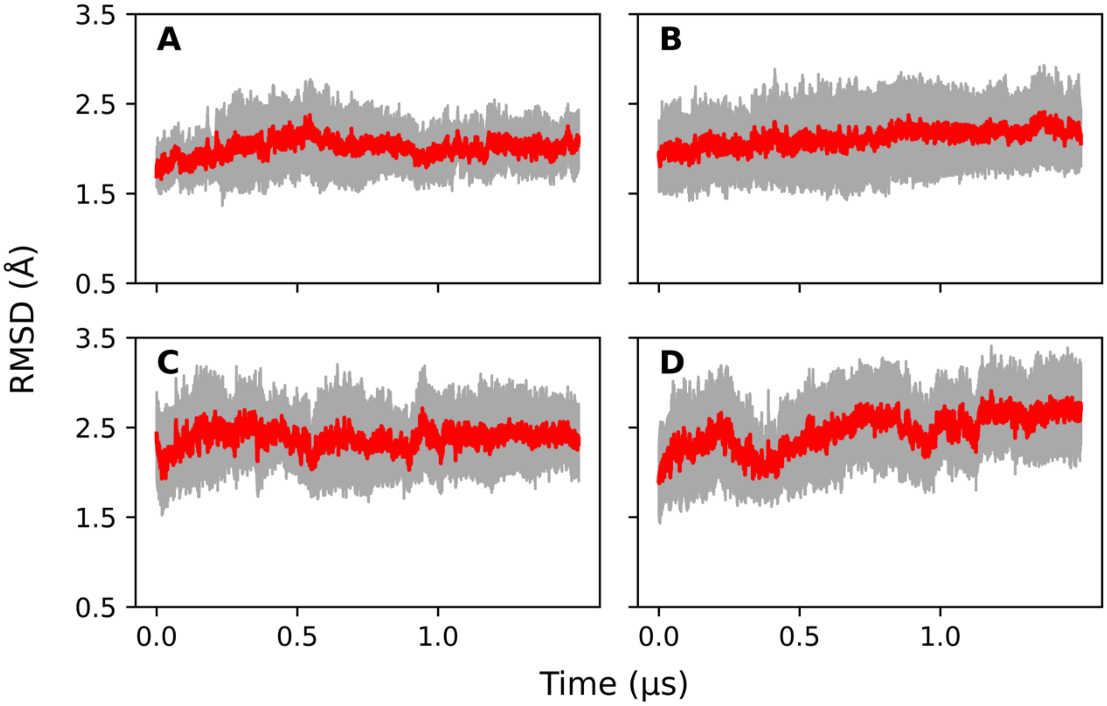

Figure S20. Root mean squared deviations (RMSD (Å)) calculated for all C_α-atoms of the catalytic domain of Q506P SHP-2, calculated from simulations initiated from the (**A**) WPD-loop open and unliganded, (**B**) WPD-loop open and phosphoenzyme intermediate, (**C**) WPD-loop closed and unliganded, and (**D**) WPD-loop closed and phosphoenzyme intermediate states. The red lines indicate averages, and gray shading indicates standard deviations calculated over 8 x 1.5μs independent MD replicas.

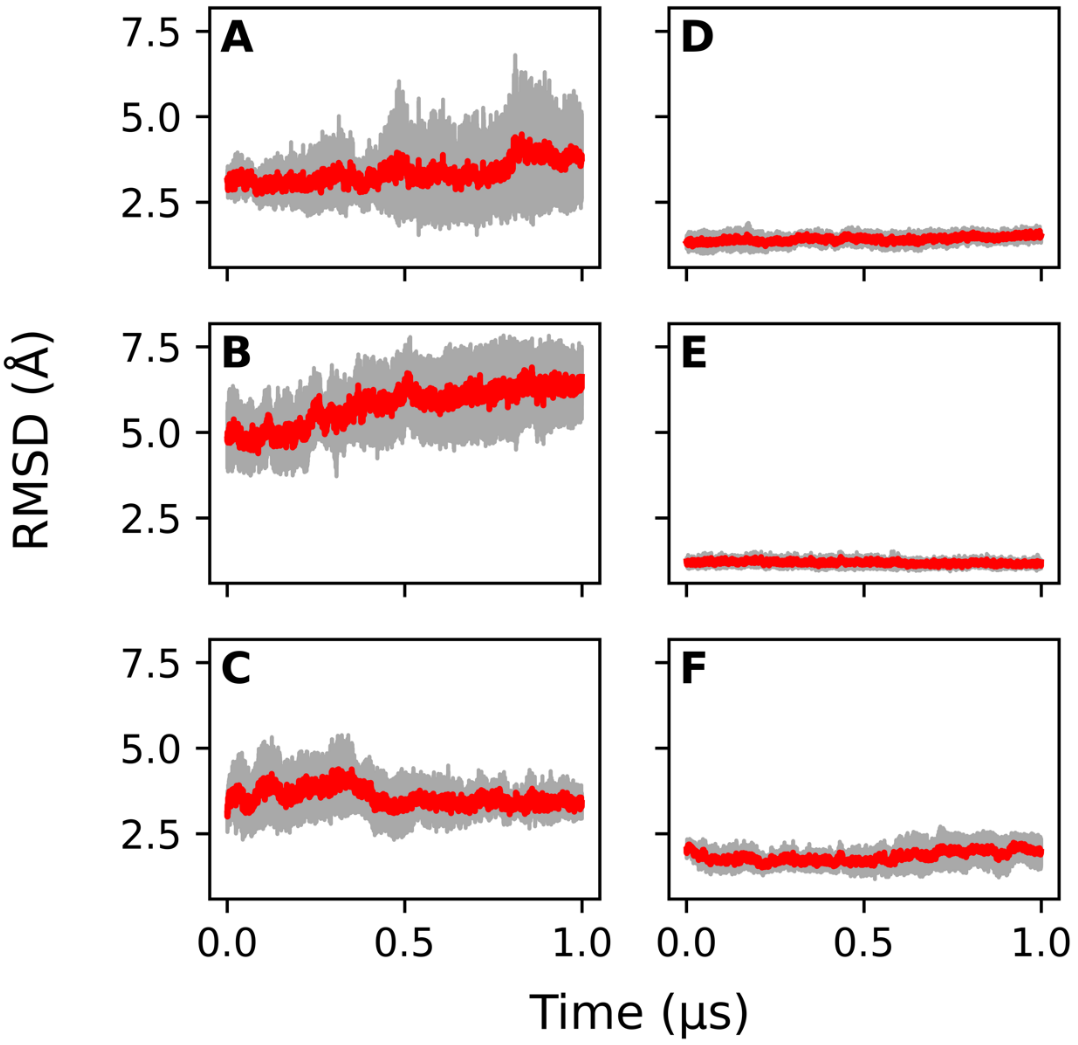

Figure S21. Root mean squared deviations (RMSD (Å)) calculated for (**A-C**) all C_α-atoms and (**D-F**) catalytic domain C_α-atoms only for **A/D**) SHP-1 open SH2 domains (PDB ID: 3PS5 [xx]), **B/E**) SHP-1 closed SH2 domains (PDB ID: 2B3O), and C/F) SHP-2 closed SH2 domains (PDB ID: 4DGP). Catalytic domain only RMSDs were calculated after alignment solely on catalytic domain atoms, and C-terminal residues 520-525 were excluded from RMSD calculations due to excessive flexibility.

.

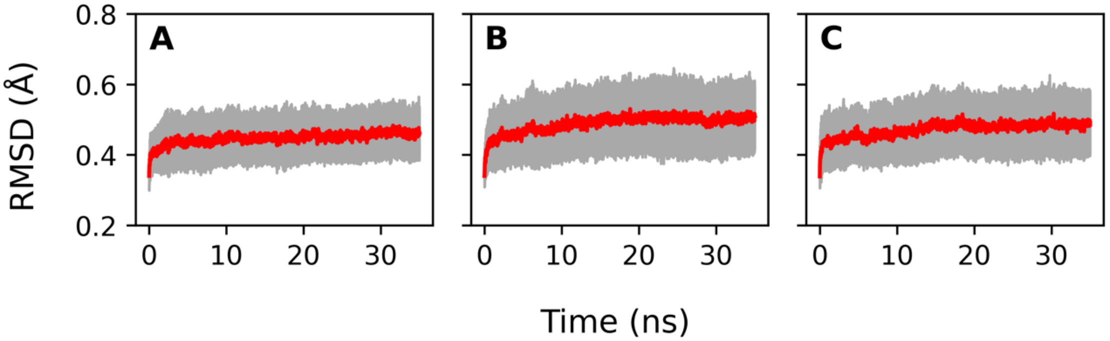

Figure S22. Root mean squared deviations (RMSD (Å)) calculated for all C_α-atoms of the catalytic domain of wild-type SHP-1 (**A**), A323T variant (**B**), and T501M variant (**C**) over the course of 35ns equilibration before initiating empirical valence bond (EVB) simulations.[6] The red lines indicate averages and gray shading indicates standard deviations calculated over 30 x 35ns independent MD replicas.

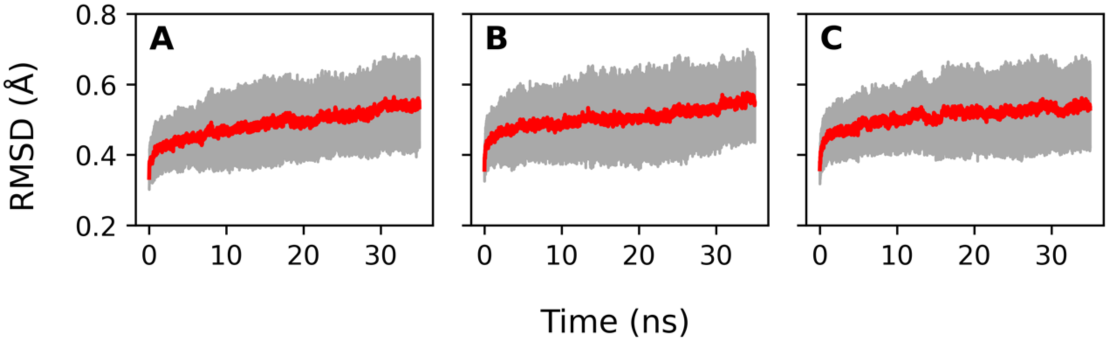

Figure S23. Root mean squared deviations (RMSD (Å)) calculated for all C_α-atoms of the catalytic domain of wild-type SHP-2 (**A**), N308D variant (**B**), and Q506P variant (**C**) over the course of 35ns equilibration before initiating empirical valence bond (EVB) simulations.[6] The red lines indicate averages and gray shading indicates standard deviations calculated over 30 x 35ns independent MD replicas.

Supplementary Tables

Table S1. Pairwise distances used for distance root mean square deviation (dRMSD) calculations.[a]

Unliganded structures:

Residue Index	Residue Index	Residue Name	Residue Name	Distance (Å)
413	452	GLN	HID	9.9
413	453	GLN	CYM	9.9
413	454	GLN	SER	12.8
413	455	GLN	ALA	15.6
413	456	GLN	GLY	14.1
413	457	GLN	ILE	16.3
413	458	GLN	GLY	15.6
414	452	TYR	HID	10.5
414	453	TYR	CYM	9.6
414	454	TYR	SER	12.5
414	455	TYR	ALA	15.2
414	456	TYR	GLY	13.9
414	457	TYR	ILE	15.3
414	458	TYR	GLY	13.8
415	452	LEU	HID	12.6
415	453	LEU	CYM	10.7
415	454	LEU	SER	12.7
415	455	LEU	ALA	15.9
415	456	LEU	GLY	15.4
415	457	LEU	ILE	17.0
415	458	LEU	GLY	15.1
416	452	SER	HID	14.5
416	453	SER	CYM	12.0
416	454	SER	SER	13.8
416	455	SER	ALA	16.7
416	456	SER	GLY	16.4
416	457	SER	ILE	17.2
416	458	SER	GLY	14.6
417	452	TRP	HID	12.3
417	453	TRP	CYM	9.6
417	454	TRP	SER	11.6
417	455	TRP	ALA	13.9
417	456	TRP	GLY	13.5
417	457	TRP	ILE	13.8
417	458	TRP	GLY	10.9
418	452	PRO	HID	13.4

418	453	PRO	CYM	10.0
418	454	PRO	SER	10.7
418	455	PRO	ALA	12.9
418	456	PRO	GLY	13.5
418	457	PRO	ILE	13.5
418	458	PRO	GLY	10.4
419	452	ASP	HID	12.0
419	453	ASP	CYM	8.4
419	454	ASP	SER	8.0
419	455	ASP	ALA	9.8
419	456	ASP	GLY	11.1
419	457	ASP	ILE	11.0
419	458	ASP	GLY	8.3
420	452	HIE	HID	13.5
420	453	HIE	CYM	10.3
420	454	HIE	SER	10.1
420	455	HIE	ALA	10.5
420	456	HIE	GLY	11.6
420	457	HIE	ILE	10.2
420	458	HIE	GLY	6.9
421	452	GLY	HID	14.7
421	453	GLY	CYM	11.7
421	454	GLY	SER	12.4
421	455	GLY	ALA	13.4
421	456	GLY	GLY	13.7
421	457	GLY	ILE	12.4
421	458	GLY	GLY	8.7
422	452	VAL	HID	15.0
422	453	VAL	CYM	12.6
422	454	VAL	SER	14.3
422	455	VAL	ALA	15.3
422	456	VAL	GLY	14.7
422	457	VAL	ILE	13.3
422	458	VAL	GLY	9.6
423	452	PRO	HID	15.2
423	453	PRO	CYM	13.2
423	454	PRO	SER	15.5
423	455	PRO	ALA	17.1
423	456	PRO	GLY	16.0
423	457	PRO	ILE	15.3
423	458	PRO	GLY	11.9
424	452	SER	HID	18.7
424	453	SER	CYM	16.6
424	454	SER	SER	18.7
424	455	SER	ALA	20.5

424	456	SER	GLY	19.7
424	457	SER	ILE	19.0
424	458	SER	GLY	15.6
425	452	GLU	HID	20.0
425	453	GLU	CYM	18.4
425	454	GLU	SER	21.0
425	455	GLU	ALA	22.4
425	456	GLU	GLY	21.0
425	457	GLU	ILE	20.0
425	458	GLU	GLY	16.7
426	452	PRO	HID	19.5
426	453	PRO	CYM	18.5
426	454	PRO	SER	21.4
426	455	PRO	ALA	22.4
426	456	PRO	GLY	20.5
426	457	PRO	ILE	19.2
426	458	PRO	GLY	16.3
427	452	GLY	HID	20.8
427	453	GLY	CYM	20.1
427	454	GLY	SER	23.2
427	455	GLY	ALA	24.6
427	456	GLY	GLY	22.6
427	457	GLY	ILE	21.8
427	458	GLY	GLY	19.1
428	452	GLY	HID	18.2
428	453	GLY	CYM	17.3
428	454	GLY	SER	20.4
428	455	GLY	ALA	22.2
428	456	GLY	GLY	20.4
428	457	GLY	ILE	20.1
428	458	GLY	GLY	17.4

Phosphoenzyme intermediate structures:

413	452	GLN	HID	9.9
413	453	GLN	CSP	9.9
413	454	GLN	SER	12.8
413	455	GLN	ALA	15.6
413	456	GLN	GLY	14.1
413	457	GLN	ILE	16.3
413	458	GLN	GLY	15.6
414	452	TYR	HID	10.5
414	453	TYR	CSP	9.6
414	454	TYR	SER	12.5
414	455	TYR	ALA	15.2

414	456	TYR	GLY	13.9
414	457	TYR	ILE	15.3
414	458	TYR	GLY	13.8
415	452	LEU	HID	12.6
415	453	LEU	CSP	10.7
415	454	LEU	SER	12.7
415	455	LEU	ALA	15.9
415	456	LEU	GLY	15.4
415	457	LEU	ILE	17.0
415	458	LEU	GLY	15.1
416	452	SER	HID	14.5
416	453	SER	CSP	12.1
416	454	SER	SER	13.8
416	455	SER	ALA	16.7
416	456	SER	GLY	16.4
416	457	SER	ILE	17.2
416	458	SER	GLY	14.6
417	452	TRP	HID	12.3
417	453	TRP	CSP	9.8
417	454	TRP	SER	11.6
417	455	TRP	ALA	13.9
417	456	TRP	GLY	13.5
417	457	TRP	ILE	13.8
417	458	TRP	GLY	10.9
418	452	PRO	HID	13.4
418	453	PRO	CSP	10.1
418	454	PRO	SER	10.7
418	455	PRO	ALA	12.9
418	456	PRO	GLY	13.5
418	457	PRO	ILE	13.5
418	458	PRO	GLY	10.4
419	452	ASP	HID	12.0
419	453	ASP	CSP	8.5
419	454	ASP	SER	8.0
419	455	ASP	ALA	9.8
419	456	ASP	GLY	11.1
419	457	ASP	ILE	11.0
419	458	ASP	GLY	8.3
420	452	HIE	HID	13.5
420	453	HIE	CSP	10.5
420	454	HIE	SER	10.1
420	455	HIE	ALA	10.5
420	456	HIE	GLY	11.6
420	457	HIE	ILE	10.2
420	458	HIE	GLY	6.9

421	452	GLY	HID	14.7
421	453	GLY	CSP	11.9
421	454	GLY	SER	12.4
421	455	GLY	ALA	13.4
421	456	GLY	GLY	13.7
421	457	GLY	ILE	12.4
421	458	GLY	GLY	8.7
422	452	VAL	HID	15.0
422	453	VAL	CSP	12.8
422	454	VAL	SER	14.3
422	455	VAL	ALA	15.3
422	456	VAL	GLY	14.7
422	457	VAL	ILE	13.3
422	458	VAL	GLY	9.6
423	452	PRO	HID	15.2
423	453	PRO	CSP	13.3
423	454	PRO	SER	15.5
423	455	PRO	ALA	17.1
423	456	PRO	GLY	16.0
423	457	PRO	ILE	15.3
423	458	PRO	GLY	11.9
424	452	SER	HID	18.7
424	453	SER	CSP	16.7
424	454	SER	SER	18.7
424	455	SER	ALA	20.5
424	456	SER	GLY	19.7
424	457	SER	ILE	19.0
424	458	SER	GLY	15.6
425	452	GLU	HID	20.0
425	453	GLU	CSP	18.6
425	454	GLU	SER	21.0
425	455	GLU	ALA	22.4
425	456	GLU	GLY	21.0
425	457	GLU	ILE	20.0
425	458	GLU	GLY	16.7
426	452	PRO	HID	19.5
426	453	PRO	CSP	18.7
426	454	PRO	SER	21.4
426	455	PRO	ALA	22.4
426	456	PRO	GLY	20.5
426	457	PRO	ILE	19.2
426	458	PRO	GLY	16.3
427	452	GLY	HID	20.8
427	453	GLY	CSP	20.2
427	454	GLY	SER	23.2

427	455	GLY	ALA	24.6
427	456	GLY	GLY	22.6
427	457	GLY	ILE	21.8
427	458	GLY	GLY	19.1
428	452	GLY	HID	18.2
428	453	GLY	CSP	17.5
428	454	GLY	SER	20.4
428	455	GLY	ALA	22.2
428	456	GLY	GLY	20.4
428	457	GLY	ILE	20.1
428	458	GLY	GLY	17.4

[a] HID and HIE refer to histidine side chains protonated at $N^{\delta 1}$ and $N^{\varepsilon 1}$, respectively. CYM and CSP refer to the charged and phosphorylated (pCys) forms of the nucleophilic cysteine, respectively. Residue indices are based on SHP-1 residue numbering.

Table S2. Conservation of allostery between different PTPs considered in this work.[a]

	**PTP1B**	**SHP-1**	**SHP-2**	**YopH**
**PTP1B**	--	42.3%	36.5%	36.5%
**SHP-1**	22/52	--	52.5%	31.1%
**SHP-2**	19/52	32/61	--	33.9%
**YopH**	19/52	19/61	20/59	--

[a] Fraction of allosteric "hotspot" residues shared between pairs of PTPs, calculated using the shortest path map (SPM)[10] approach, as described in the main text. Fractions are given with respect to the total number of SPM residues at the given column, and percentages are calculated from the corresponding fractions.

Table S3. SPM[11]-classified oncogenic variants as obtained from the COSMIC[12] database.[a]

	**SHP-1**	**SHP-2**
**SPM pathway**	323, 326, 393, 501, 505	265, 288, 308, 309, 414, 454, 498, 505, 506, 507, 510
**≤4Å from path**	262, 275, 339, 352, 362, 375, 388 419, 459, 462, 513	262, 278, 282, 285, 293, 306, 311, 347, 351, 357, 397, 411, 419, 426, 428, 438, 461, 468, 499, 500, 502, 503, 511, 512
**>4Å from path**	243, 316, 317, 353, 355, 366, 378, 382, 399,	279, 343, 381, 382, 384, 391, 409, 425, 433, 437, 444, 483, 489, 491, 527

[a] Only residue positions are noted, since there are often many mutations on the same positions which are marked as separate COSMIC entries.

Table S4. Experimental and calculated activation free energies, and corresponding experimental turnover numbers, for wild-type and variant forms of SHP-1 and SHP-2.[a]

	$\Delta G^{\ddagger}_{calc}$	$\Delta G^{\ddagger}_{exp}$	k_{cat}
SHP-1			
SHP-1 WT	15.1 ± 0.3	16.3 (pH 7.4), 14.2-15.1 (pH 5.0)	5.7 (pH 7.4),[13] 57.6-200.9 (pH 5.0)[13-16]
A323T	17.0 ± 0.4	—	—
T501M	16.3 ± 0.3	—	—
SHP-2			
SHP-2 WT	15.7 ± 0.3	16.4 (pH 7.0), 16.1 (pH 7.5), 15.7-16.1 (pH 5.0)	6.4 (pH 7.0)[17], 9.26 (pH 7.5),[18] 15.9-70.4 (pH 5.0)[19]
SHP-2 N308D	17.1 ± 0.3	15.9 (pH 7.5)	12.82 (pH 7.5)[18]
SHP-2 Q506P	15.9 ± 0.3	17.7 (pH 7.0,7.5)	0.61 (pH 7.5),[18] 0.64 (pH 7.0)[17]

[a] References to experimental kinetic data are provided in the table. The k_{cat} values are presented in s^{-1}, and all energies are presented in kcal mol^{-1}. All calculated activation free energies ($\Delta G^{\ddagger}$) are presented as average values and standard error of the mean over 30 independent EVB simulations. "—" indicates experimental data not available. Note that as in some cases, kinetics have been measured under a range of experimental conditions, as indicated in the table.

Table S5. Calculated distances at the phosphoenzyme intermediate (RS), transition states (TS), and product states (PS) as obtained from our EVB simulations.[a]

System	State	S_{cys}-P	P-O_{H2O}	O_{H20}-H	H-O_{Asp}
SHP-1 WT	RS	2.0±0.0	3.2±0.2	1.0±0.0	3.0±0.8
	TS	2.5±0.6	2.1±0.2	1.2±0.2	1.2±0.1
	PS	3.2±0.0	1.6±0.0	1.3±0.0	1.0±0.0
SHP-2 A323T	RS	2.0±0.0	3.4±0.3	1.0±0.0	2.6±0.9
	TS	2.6±0.7	2.0±0.2	1.2±0.3	1.2±0.1
	PS	3.3±0.1	1.6±0.0	1.3±0.0	1.0±0.0
SHP-1 T501M	RS	2.0±0.0	3.6±0.6	1.0±0.0	2.4±1.0
	TS	2.6±0.5	2.0±0.2	1.2±0.2	1.2±0.1
	PS	3.3±0.1	1.6±0.0	1.3±0.0	1.0±0.0
SHP-2 WT	RS	2.0±0.0	3.7±0.8	1.0±0.0	2.8±1.0
	TS	2.4±0.2	2.2±0.1	1.2±0.4	1.2±0.0
	PS	3.3±0.1	1.6±0.0	1.3±0.0	1.0±0.0
SHP-2 N308D	RS	2.0±0.0	3.5±0.5	1.0±0.0	2.8±1.1
	TS	2.5±0.4	2.1±0.1	1.1±0.1	1.2±0.1
	PS	3.3±0.1	1.6±0.0	1.3±0.0	1.0±0.0
SHP-2 Q506P	RS	2.0±0.0	3.5±0.6	1.0±0.0	2.8±1.1
	TS	2.4±0.2	2.1±0.1	1.1±0.1	1.2±0.0
	PS	3.3±0.2	1.6±0.0	1.3±0.0	1.0±0.0

[a] Distances are measured in Å and are shown as average values ± standard deviation over 30 independent EVB trajectories for each system.

Table S6. Overview of crystal structures used in this work.

PDB ID	Protein	WPD-loop Conformation	Domains
4GRZ[8]	SHP-1	Closed (unliganded and intermediate)	Catalytic domain
4HJP[9]	SHP-1	Open (unliganded and intermediate)	Catalytic domain
6CMQ[20]	SHP-2	Closed/Open (unliganded and intermediate)	Catalytic domain (NSH2 removed)
3PS5[1]	SHP-1	Open (unliganded)	Catalytic domain+N-SH2+C-SH2 (open)
2B3O[2]	SHP-1	Open (unliganded)	Catalytic domain+N-SH2+C-SH2 (autoinhibited)
4DGP[21]	SHP-2	Open (unliganded)	Catalytic domain+N-SH2+C-SH2 (autoinhibited)
6B90[4]	PTP1B	Closed/Open (unliganded and open intermediate)	Catalytic domain
3I80[5]	PTP1B	Closed (intermediate)	Catalytic domain
2I42[22]	YopH	Closed (unliganded and intermediate)	Catalytic domain
1YPT[23]	YopH	Open (unliganded)	Catalytic domain

Table S7. Ionization residues and histidine protonation patterns in the empirical valence bond (EVB) simulations of SHP-1 and SHP-2.[a]

Residue Type	SHP-1	SHP-2
Asp	334, 386, 419, 434	395, 425, 431, 473, 477
Glu	249, 255, 329, 353, 355, 384, 389, 425, 502	258, 359, 361, 390
Arg	262, 275, 292, 352, 358, 393, 459, 492, 495	278, 362, 399, 421, 465, 498, 501
Lys	273, 277, 356, 360, 391, 506	325, 358, 364, 366
His-δ	411, 452	287, 443, 458, 520
His-ε	260, 284, 385, 420, 445	293, 394, 419, 426

[a] All other residues were kept in their neutral state, as they fell outside the explicit simulation sphere using the surface constraint all atom solvent model (SCAAS).[24] Equivalent ionization states were used for all oncogenic variants.

Supplementary References

(1) Wang, W.; Liu, L.; Song, X.; Mo, Y.; Komma, C.; Bellamy, H. D.; Zhao, Z. J.; Zhou, G. W. Crystal Structure of Human Protein Tyrosine Phosphatase SHP-1 in the Open Conformation. *J. Cell. Biochem.* **2011**, *112*, 2062–2071.

(2) Yang, J.; Liu, L.; He, D.; Song, X.; Liang, X.; Zhao, Z. J.; Zhou, G. W. Crystal Structure of Human Protein-Tyrosine Phosphatase SHP-1. *J. Biol. Chem.* **2003**, *278*, 6516–6520.

(3) Edgar, R. C. MUSCLE: A Multiple Sequence Alignment With High Accuracy and High Throughput. *Nucleic Acids Res.* **2004**, *32*, 1792–1797.

(4) Keedy, D. A.; Hill, Z. B.; Biel, J. T.; Kang, E.; Rettenmaier, T. J.; Brandão-Neto, J.; Pearce, N. M.; von Delft, F.; Wells, J. A.; Fraser, J. S. An Expanded Allosteric Network in PTP1B by Multitemperature Crystallography, Fragment Screening, and Covalent Tethering. *eLife* **2018**, *7*, 1–36.

(5) Brandão, T. A. S.; Hengge, A. C.; Johnson, S. J. Insights into the Reaction of Protein-Tyrosine Phosphatase 1B. *J. Biol. Chem.* **2010**, *285*, 15874–15883.

(6) Warshel, A.; Weiss, R. M. An Empirical Valence Bond Approach for Comparing Reactions in Solutions and in Enzymes. *J. Am. Chem. Soc.* **1980**, *102*, 6218–6226.

(7) Roe, D. R.; Cheatham, T. E. PTRAJ and CPPTRAJ: Software for Processing and Analysis of Molecular Dynamics Trajectory Data. *J. Chem. Theory. Comput.* **2013**, *9*, 3084–3095.

(8) Alicea-Velázquez, N. L.; Jakoncic, J.; Boggon, T. J. Structure-Guided Studies of the SHP-1/JAK1 Interaction Provide New Insights Into Phosphatase Catalytic Domain Substrate Recognition. *J. Struct. Biol.* **2013**, *181*, 243–251.

(9) Alicea-Velazquez, N. L.; Boggon, T. J. SHP Family Protein Tyrosine Phosphatases Adopt Canonical Active-Site Conformations in the Apo and Phosphate-Bond States. *Protein Pept. Lett.* **2013**, *20*, 1039–1048.

(10) Romero-Rivera, A.; Garcia-Borras, M.; Osuna, S. Role of Conformational Dynamics in the Evolution of Retro-Aldolase Activity. *ACS Catal.* **2017**, *7*, 8524–8532.

(11) Maria-Solano, M. A.; Serrano-Hervás, E.; Romero-Rivera, A.; Iglesias-Fernández, J.; Osuna, S. Role of Conformational Dynamics in the Evolution of Novel Enzyme Function. *Chem. Commun.* **2018**, *54*, 6622–6634.

(12) Tate, J. G.; Bamford, S.; Jubb, H. C.; Sondka, Z.; Beare, D. M.; Bindal, N.; Boutselakis, H.; Cole, C. G.; Creatore, C.; Dawson, E.; et al. COSMIC: The Catalogue of Somatic Mutations in Cancer. *Nucleic Acids Res.* **2018**, *47*, D941–D947.

(13) Pei, D.; Lorenz, U.; Klingmüller, U.; Neel, B. G.; Walsh, C. T. Intramolecular Regulation of Protein Tyrosine Phosphatase SH-PTP1: A New Function for Src Homology 2 Domains. *Biochemistry* **1994**, *33*, 15483–15493.

(14) Yang, J.; Liang, X.; Niu, T.; Meng, W.; Zhao, Z.; Zhou, G. W. Crystal Structure of the Catalytic Domain of Protein-Tyrosine Phosphatase SHP-1. *J. Biol. Chem.* **1998**, *273*, 28199–28207.

(15) Yang, J.; Niu, T.; Zhang, A.; Mishra, A. K.; Zhao, Z. J.; Zhou, G. W. Relationship Between the Flexibility of the WPD Loop and the Activity of the Catalytic Domain of Protein Tyrosine Phosphatase SHP-1. *J. Cell. Biochem.* **2001**, *84*, 47–55.

(16) Taga, M. E.; Larsen, N. A.; Howard-Jones, A. R.; Walsh, C. T.; Walker, G. C. BluB Cannibalizes Flavin to Form the Lower Ligand of Vitamin B12. *Nature* **2007**, *446*, 449–453.

(17) Yu, Z.-H.; Zhang, R.-Y.; Walls, C. D.; Chen, L.; Zhang, S.; Wu, L.; Liu, S.; Zhang, Z.-Y. Molecular Basis of Gain-of-Function LEOPARD Syndrome-Associated SHP2 Mutations. *Biochemistry* **2014**, *53*, 4136–4151.

(18) Qiu, W.; Wang, X.; Romanov, V.; Hutchinson, A.; Lin, A.; Ruzanov, M.; Battaile, K. P.; Pai, E. F.; Neel, B. G.; Chirgadze, N. Y. Structural Insights into Noonan/LEOPARD Syndrome-Related Mutants of Protein-Tyrosine Phosphatase SHP2 (PTPN11). *BMC Struct. Biol.* **2014**, *14*, 10.

(19) Niu, T.; Liang, X.; Yang, J.; Zhao, Z.; Zhou, G. W. Kinetic Comparison of the Catalytic Domains of SHP-1 and SHP-2. *J. Cell Biochem.* **1999**, *72*, 145–150.

(20) Padua, R. A. P.; Sun, Y.; Marko, I.; Pitsawong, W.; Stiller, J. B.; Otten, R.; Kern, D. Mechanism of Activating Mutations and Allosteric Drug Inhibition of the Phosphatase SHP2. *Nat. Commun.* **2018**, *9*, 4507.

(21) Yu, Z. H.; Xu, J.; Walls, C. D.; Chen, L.; Zhang, S.; Zhang, R. Y.; Wu, L.; Wang, L. N.; Liu, S. J.; Zhang, Z.-Y. Structural and Mechanistic Insights into LEOPARD Syndrome-Associated SHP2 Mutations. *J. Biol. Chem.* **2013**, *288*, 10472–10482.

(22) Berman, H. M.; Westbrook, J.; Feng, Z.; Gilliland, G.; Bhat, T. N.; Weissig, H.; Shindyalov, I. N.; Bourne, P. E. The Protein Data Bank. *Nucleic Acids Res.* **2000**, *28*, 235–242.

(23) Stuckey, J. A.; Schubert, H. L.; Fuaman, E. B.; Zhang, Z.-Y.; Dixon, J. E.; Saper, M. A. Crystal Structure of Yersinia Protein Tyrosine Phosphatase at 2.5 Å and the Complex with Tungstate. *Nature* **1994**, *370*, 571–575.

(24) Warshel, A.; King, G. Polarization Constraints in Molecular Dynamics Simulation of Aqueous Solutions: The Surface Constraint All Atom Solvent (SCAAS) Model. *Chem. Phys. Lett.* **1985**, *121*, 124–129.
